# The plasma membrane H^+^-ATPase gene family in *Solanum tuberosum* L. Role of PHA1 in tuberization

**DOI:** 10.1093/jxb/erx284

**Published:** 2017-08-19

**Authors:** Margarita Stritzler, María Noelia Muñiz García, Mariana Schlesinger, Juan Ignacio Cortelezzi, Daniela Andrea Capiati

**Affiliations:** 1 Institute of Genetic Engineering and Molecular Biology ‘Dr. Héctor Torres’ (INGEBI), National Research Council (CONICET), Vuelta de Obligado, Buenos Aires, Argentina; 2 Biochemistry Department, School of Exact and Natural Sciences, University of Buenos Aires, Buenos Aires, Argentina

**Keywords:** Branching, PHA1, plant growth, PM H^+^-ATPase, potato, stolon elongation, tuber growth, tuberization

## Abstract

This study presents the characterization of the plasma membrane (PM) H^+^-ATPases in potato, focusing on their role in stolon and tuber development. Seven PM H^+^-ATPase genes were identified in the *Solanum tuberosum* genome, designated *PHA1–PHA7*. PHA genes show distinct expression patterns in different plant tissues and under different stress treatments. Application of PM H^+^-ATPase inhibitors arrests stolon growth, promotes tuber induction, and reduces tuber size, indicating that PM H^+^-ATPases are involved in tuberization, acting at different stages of the process. Transgenic potato plants overexpressing *PHA1* were generated (PHA1-OE). At early developmental stages, PHA1-OE stolons elongate faster and show longer epidermal cells than wild-type stolons; this accelerated growth is accompanied by higher cell wall invertase activity, lower starch content, and higher expression of the sucrose–H^+^ symporter gene *StSUT1*. PHA1-OE stolons display an increased branching phenotype and develop larger tubers. PHA1-OE plants are taller and also present a highly branched phenotype. These results reveal a prominent role for PHA1 in plant growth and development. Regarding tuberization, PHA1 promotes stolon elongation at early stages, and tuber growth later on. PHA1 is involved in the sucrose–starch metabolism in stolons, possibly providing the driving force for sugar transporters to maintain the apoplastic sucrose transport during elongation.

## Teaser

Genetic evidence is provided for the role of potato PM H^+^-ATPases in tuberization, demonstrating that the isoform PHA1 promotes stolon elongation and tuber growth, and is involved in sugar–starch metabolism.

## Introduction

Plasma membrane (PM) H^+^-ATPases are integral membrane proteins that pump protons out of the cell, generating an electrochemical gradient of protons across the plasmalemma. As a result, PM H^+^-ATPases provide the driving force for the transport of ions and metabolites through channels and transporters (Palmgren, 2001). This process is required for several physiological responses, such as cell expansion, phloem loading/unloading, stress adaptation, and plant growth and development (Michelet and Boutry, 1995; Palmgren, 2001; Gaxiola *et al.*, 2007; Duby and Boutry, 2009; Schumacher and Krebs, 2010). PM H^+^-ATPases have been described in many plant species, such as *Arabidopsis thaliana* (Gaxiola *et al.*, 2007), *Nicotiana plumbaginifolia* (Morsomme and Boutry, 2000), *Oryza sativa* (Baxter *et al.*, 2003), *Solanum lycopersicum* (Kalampanayil and Wimmers, 2001), *Zea mays* (Santi *et al.*, 2003), and *Cucumis sativus* (Wdowikowska and Klobus, 2016).

The plant PM H^+^-ATPases are regulated at different levels. With respect to post-translational regulation, the mechanism involves the autoinhibitory action of the C-terminal domain that can be displaced by phosphorylation of the penultimate residue, a threonine, and the subsequent binding of 14-3-3 proteins, resulting in pump activation (Palmgren *et al.*, 1991; Svennelid *et al.*, 1999; Maudoux *et al.*, 2000). More recently, it has been described that phosphorylation of other residues can also affect PM H^+^-ATPase activity (Duby *et al.*, 2009; Piette *et al.*, 2011), suggesting that these proton pumps are controlled by a complex regulatory mechanism. Other factors can regulate PM H^+^-ATPase activity. A novel interaction partner of PM H^+^-ATPase named PPI (proton pump interactor) was identified in *A. thaliana* (PPI1; Morandini *et al.*, 2002) and *Solanum tuberosum* (StPPI1; Muñiz García *et al.*, 2011); this protein increases the activity of the proton pump *in vitro* once the C-terminus has been displaced.

Several studies have indicated that regulation of PM H^+^-ATPase also occurs at the genetic level. PM H^+^-ATPases are encoded by five distinct gene subfamilies. The members of subfamilies I and II show ubiquitous expression profiles, while the expression of genes belonging to subfamilies III, IV, and V is restricted to specific tissues (Arango *et al.*, 2003; Gaxiola *et al.*, 2007). PM H^+^-ATPase transcript levels are differentially regulated by environmental and hormonal signals, such as salt stress (Kalampanayil and Wimmers, 2001; Janicka-Russak and Kłobus, 2007; Sahu and Shaw, 2009), dehydration (Surowy and Boyer, 1991), low temperature (Ahn *et al.*, 1999), and exogenous application of hormones (Frías *et al.*, 1996; Janicka-Russak and Kłobus, 2007).

In potato (*Solanum tuberosum* cv. Désirée), two genes encoding PM H^+^-ATPases (*PHA1* and *PHA2*) were isolated long ago by Harms *et al.* (1994). However, their physiological functions have not yet been elucidated. The economic importance of potato plants resides in the capacity to produce tubers. Potato tubers develop from stolons, which are underground stems. All aspects of tuber development are co-ordinated by a complex interaction of phytohormones and environmental signals (Sarkar, 2008). Gibberellic acid (GA) is a central negative regulator of tuberization that stimulates stolon elongation and prevents tuber induction (Xu *et al.*, 1998). Abscisic acid (ABA) and auxin also regulate tuberization, acting as promoting factors (Xu *et al.*, 1998; Roumeliotis *et al.*, 2012; Kolachevskaya *et al.*, 2015).

At early stages of tuber formation, stolons display a cessation of elongation and the initiation of subapical radial growth, which is followed by a deposition of starch and storage proteins (Visser *et al.*, 1994). During the transition from stolon to tuber, a switch from apoplastic to symplastic sucrose phloem unloading occurs (Viola *et al.*, 2001). Phloem loading of sucrose in source leaves requires the activity of sucrose–H^+^ symporters, and apoplastic sucrose unloading in sink tissues involves sucrose–H^+^ and hexose–H^+^ symporters (Lemoine *et al.*, 2013). These sugar transporters depend on the proton motive force generated by PM H^+^-ATPases; therefore, these proton pumps might play an important role in stolon and tuber development.

This study presents a comprehensive overview of the PM H^+^-ATPase gene family (PHA) in potato, including the identification of PHA genes and expression profiles to explore which PHA genes are possibly involved in tuberization. Based on the results of the expression analysis, *PHA1* was chosen for further characterization by the development of transgenic plants.

## Materials and methods

### Plant material

Soil-grown potato plants (*Solanum tuberosum* cv. Spunta) were cultivated in a growth chamber at 19 °C or 22 °C, under a 12 h or 16 h light photoperiod (4000 lux light intensity), or in a greenhouse maintained between 22 °C and 24 °C, under a 16 h light photoperiod. *In vitro* plants were obtained by micropropagation of virus-free single-node cuttings in Murashige and Skoog medium (MS medium; Prod no. M519, PhytoTechnology Laboratories, Shawnee Mission, KS, USA) containing 20 g l^–1^ sucrose solidified with 0.7% (w/v) agar, under a 16 h light photoperiod (4000 lux light intensity) at 22 °C.

### Abiotic stress treatments

The first two fully expanded leaves detached from plants grown in soil, in a greenhouse, for 4 weeks were used for salt stress and drought treatments. Prior to stress treatment, leaves were placed in individual containers with water in a growth chamber at 22 °C, under a 16 h light photoperiod, for 48 h, to allow the wound response components induced by excision to be restored to basal levels. Only the cut end of the petioles was immersed in the solution. For salt stress, leaves were treated with 250 mM NaCl. For drought treatment, water was removed from the containers and leaves were kept in air. For cold treatment, *in vitro* grown potato plants were exposed to 4 °C, while control plants remained at 22 °C, under a 16 h light photoperiod.

### Stolon growth conditions and *in vitro* tuberization

Tuberization can be studied *in vitro*, reproducing the process occurring *in vivo* with the advantages of generating tubers in a controlled environment (Xu *et al.*, 1998; Roumeliotis *et al.*, 2012; Muñiz García *et al.*, 2016). Single-node cuttings obtained from potato plants do not induce tubers when cultured in darkness on standard propagation media (MS medium plus 2% sucrose); however, increasing sucrose concentration (8%) increases the frequency of tuberization.

Shoot apices derived from *in vitro* plants were cultured on solid MS media containing 20 g l^–1^ sucrose for 2 weeks, in a growth chamber at 22 °C, under a 16 h light photoperiod, prior to harvesting single-node explants. Nodal segments were grown on MS medium containing 20 g l^–1^ or 80 g l^–1^ sucrose (non-inducing and tuber-inducing conditions, respectively) solidified with 0.7% (w/v) agar, in a growth chamber in darkness at 19 °C. In some experiments, the media was supplemented with hormones (5 μM GA_3_, 25 μM 3-indoleacetic acid, or 5 μM ABA; purchased from Sigma, St Louis, MO, USA) or the inhibitors of H^+^-ATPase (1 mM sodium orthovanadate or 50 μM erythrosine B; purchased from Sigma). The single-node cuttings formed etiolated shoots/stolons that, under tuber-inducing conditions, developed tubers. For tuber-inducing conditions, the process was studied for 9–10 weeks. After this time of culture, most of the tubers were fully developed, and the rate of tuber growth decreased significantly.

All the experiments were performed at least three times independently, using 10–20 stolons per condition. A tuberizing stolon was defined as a stolon presenting visible subapical swelling or tubers. Stolons presenting more than one subapical swelling/tuber were considered as one tuberizing stolon. A branched stolon was defined as a stolon presenting at least one branch ≥5 mm. The total stolon length was determined as the sum of the primary and secondary stolon lengths. Tuber weight was calculated as the sum of the weight of all the tubers divided by number of tubers obtained. If the stolon presented more than one tuber, only the fully developed tubers (minor diameter ≥4 mm) were used for weight determination, since, in most cases, the additional tubers were very small or undeveloped.

### Tuberization in soil-grown plants

Yield determination was carried out on plants transferred to soil *ex vitro* and cultivated in a growth chamber at 19 °C, under a 12 h light photoperiod, for 10 weeks, in 0.5 liter pots with commercial soil mixture (Grow Mix Multipro, Terrafertil Argentina). Stolon length determination was carried out on plants obtained from seed tubers, grown in 0.8 liter pots with commercial soil mixture, in greenhouse, for 4 weeks.

### Semi-quantitative reverse transcription–PCR (RT–PCR)

Semi-quantitative RT–PCR to determine PHA gene expression was performed as described in País *et al.* (2010) using the primers and reaction conditions shown in Supplementary Table S1 at *JXB* online. RT–PCR bands were quantified relative to the elongation factor 1-α gene (*EF1-α*) using ImageJ software (http://rsb.info.nih.gov/ij/).

### Real-time quantitative RT–PCR (RT–qPCR) analysis

Relative expression of *PHA1-3* and *StSUT1* was determined by RT–qPCR. RNA was isolated and cDNA synthesis was performed as described in País *et al*. (2010). The cDNA was used as template for PCR amplification. The potato *EF1-α* gene was used as reference gene using the primers forward, ATTGGAAACGGATATGCTCCA; and reverse, TCCTTACCTGAACGCCTGTCA. The primer sequences for *PHA1–PHA3* were PHA1 5' UTR forward, GGAAGAGAGGAAATTGAGAAAGATG; and PHA1 5' UTR reverse, CTCCTCTAGTTTGTTGTACCC (PCR efficiency: 2.04); PHA2 forward, AGAAAAGAAGAGACACACAAGC; and PHA2 reverse, GACACAATCCCTTTCAATGG (PCR efficiency: 1.97); PHA3 3' UTR forward, GTTGGTGTTGTGATGAGAGCG; and PHA3 3' UTR reverse, GAAGGCTCCAGGAAACAGC (PCR efficiency: 2.02). The primer sequences for *StSUT1* were obtained from Krügel *et al.* (2012) (LC-SUT1 fw, TTCCATAGCTGCTGGTGTTC; and LC-SUT1 rev, TACCAGAAATGGGTCCACAA). Reactions were performed in a final volume of 20 μl containing 4 μl of 5XHOT FIREPol EvaGreen qPCR Mix Plus (Solis-BioDyne, Tartu, Estonia). The amount of cDNA used in each reaction was derived from 1 ng of total RNA for *EF1-α*, 10 ng for *PHA1–PH3*, and 5 ng for *StSUT1* (each cDNA sample was diluted accordingly). Reactions for *EF1-α* amplification were carried out under the following conditions: 50 °C/2 min (1 cycle); 95 °C/15 min (1 cycle); 95 °C/15 s; 60 °C/1 min; 72 °C/30 s (35–40 cycles). For *PHA1–PHA3*, reactions were carried out as follows: 95 °C/15 min (1 cycle); 94 °C/2 min (1 cycle); 58 °C/1 min (1 cycle); 72 °C/1 min (1 cycle); 94 °C/30 s, 58 °C/30 s; 72 °C/45 s (35–40 cycles). For *StSUT1*, reactions were performed with the following program: 95 °C/15 min (1 cycle); 95 °C/30 s, 61 °C/30 s, 72 °C/30 s (35–45 cycles). Ampliﬁcation of a single product of the correct size for each gene was conﬁrmed by agarose gel electrophoresis. The relative expression level was calculated using the 2^–ΔΔCt^ (cycle threshold) method (Livak and Schmittgen, 2001).

### Cloning of the *PHA1* gene from *Solanum tuberosum* cv. Spunta

The *PHA1* coding sequence was obtained by RT–PCR from RNA isolated from potato (*S. tuberosum* cv. Spunta) flower bud using the primers PHA1 5'UTR Fw, GGAAGAGAGGAAATTGAGAAAGATG; and PHA1 3'UTR Rv, GCCGATAATGAATGCTGTTATAG. The amplified product was cloned into the pCR-Blunt II-TOPO vector (Thermo Fisher Scientific, Waltham, MA, USA), for sequencing.

### Yeast complementation assay

The complementation assay was carried out using the *Saccharomyces cerevisiae* strain YAK2 (*MAT*α, *ade 2-101, leu2*Δ*1, his3*-Δ*200, ura3-52, trp1*Δ*63, lys2-801 pma1*Δ*::HIS3, pma2*-Δ*::TRP1*) lacking the genomic copies of *PMA1* and *PMA2*, the two endogenous H^+^-ATPase genes. Survival is possible by the expression of the *PMA1* gene under the *GAL1* promoter on a *URA3*-bearing centromeric plasmid (de Kerchove d’Exaerde *et al.*, 1995). The YEplac181 plasmid (bearing the 2μ origin of replication and the *LEU2* marker) containing the promoter region of the yeast *PMA1* gene was used to express the different plant PM H^+^-ATPase genes. Colony PCR was performed to detect the presence of the corresponding plasmids in the YAK2 transformants. The transformed YAK2 strains were grown at 30 °C in a synthetic medium containing 0.7% yeast nitrogen base without amino acids, supplemented with all of the amino acids except those used for selection (His, Leu, Ura and Trp), and 2% glucose (MGlu-His, Leu, Ura, Trp) or 2% galactose/1% raffinose (MGal-His, Leu, Ura, Trp). Solid media contained 2% agar. The media were supplemented with 20 mM K_2_HPO_4_ and buffered to a pH of 6.5. In addition, the YAK2 transformants were grown on MGlu-His, Leu, Trp medium at pH 6.5, containing 0.1% 5-fluoro-orotic acid (5-FOA) to counter-select the plasmid bearing the yeast PM H^+^-ATPase gene.

### Generation of PHA1-OE transgenic potato plants

The *PHA1* sequence was subcloned into the pBI121 binary vector downstream of the *Cauliflower mosaic virus* 35S promoter (35S CaMV). Transformation of potato discs was performed using *Agrobacterium tumefaciens* strain LBA4404 as described in Muñiz García *et al.* (2014). Regenerated plants carrying no plasmid but obtained from the same explants and by the same regeneration method were used as controls (wild type). All plants were obtained from the same tuber; therefore, regeneration controls and transgenic lines have the same genetic background. Three independent transgenic lines (L1, L2, and L3) were selected and characterized in this study.

PCR analysis was performed to confirm the presence of the transgene using genomic DNA as template and the primers PHA1 5' UTR forward (GGAAGAGAGGAAATTGAGAAAGATG) and tNOS reverse (TGATAATCATCGCAAGACCG) to amplify the transgene but not the endogenous *PHA1* gene. PCR was also performed using primers to detect the *nptII* gene: nptII Fw, ATGATTGAAGAAGATGGATTG; and nptII Rv, GAAGAACTCGTCAAGAAGGCG. The *18S* rRNA gene was used as a positive control utilizing the primers 18S forward, GGGCATTCGTATTTCATAGTCAGAG, and 18S reverse, CGGTTCTTGATTAATGAAAACATCCT.

### PM H^+^-ATPase activity

PM H^+^-ATPase activity was determined in purified membranes isolated by a two-step aqueous two-phase partitioning system as described in Olivari *et al.* (2000) from leaves of potato plants grown in soil for 60 d or stolons cultured *in vitro* on MS medium plus 8% sucrose for 3 weeks. The PM H^+^-ATPase activity was determined in 5 mM ATP, 5 mM MgCl_2_, and 10 mM PIPES pH 7.3, with the addition of 5 mM NaN_3_, 0.1 mM sodium molybdate, and 100 mM KNO_3_, in the absence or presence of 100 μM Na_3_VO_4_. The assay was performed in the presence of 0.01 mg ml^–1^ lysophosphatidylcholine (Papini and De Michelis, 1997). The assay was carried out using the plasma membrane samples (2.5 μg of protein) in a final reaction volume of 150 µl for 30 min at 30 °C. Released Pi was measured using the malachite green method (Hohenwallner and Wimmer, 1973). The PM H^+^-ATPase activity was determined as the difference between the activity measured in the absence and presence of vanadate.

### Observation of stolon epidermal cell imprints

A thick layer of clear nail polish was brushed onto the epidermis of each stolon. Once dried, the nail polish was peeled off, placed on a clear glass, and observed using a light microscope (Olympus BX41, Olympus Optical Co. Ltd, Tokyo, Japan). Photomicrographs of the imprints were obtained at ×200 magnification in the microscope.

### Starch content

The samples (50 mg) were ground to a fine powder in liquid nitrogen and extracted three times with 10 ml of 80% ethanol, by boiling the samples in a 90 °C water bath for 20 min to dissolve sugars, dextrins, and tannins, and centrifuged at 1500 *g* for 5 min. The residues were dried, resuspended in 15 ml of 1 M HCl, and incubated at 99 °C for 45 min for starch hydrolysis. The contents were filtered through Whatman No. 40 filter paper. The filtrates were adjusted to pH 7.0 with NaOH and analyzed for glucose by the Somogyi–Nelson method (Nelson, 1994). Starch content was estimated by multiplying the glucose content by the glucose equivalent of 0.9. Results were expressed as g starch 100 g^–1^ FW.

### Cell wall invertase activity

The pellet mix procedure was used to assess the activities of cell wall invertase (Albertson *et al.*, 2001). About 50 mg of stolon tissue was homogenized in ice-cold extraction buffer at a 1:5 (w/v) ratio containing 50 mM HEPES (pH 7.5), 10 mM MgCl_2_, 1 mM EDTA, 1 mM EGTA, 1 mM benzamidine, 1 mM phenylmethylsulfonyl fluoride (PMSF), 2 μg ml^–1^ trypsin inhibitor, 5 mM aminocaproic acid, 5 mM DTT, 0.1% (v/v) Triton X-100, and 2% (w/v) polyvinylpyrrolidone (PVP). The extract was centrifuged at 12 000 *g* for 10 min at 4 °C; the pellet was washed three times with extraction buffer without PVP and used for activity assay after a final resuspension in a 1:4 (w/v) ratio with the same buffer. The activities were assayed in a final volume of 1 ml, containing 125 mM Na-acetate buffer (pH 4.5), 100 mM sucrose, and 80 μl of the pellet mix. The reactions were incubated at 37 °C for 30 min. Reactions were stopped in boiling water for 1 min, centrifuged at 12 000 *g* for 5 min, and glucose was quantified in the supernatant according to the Nelson–Somogyi method (Nelson, 1994).

### Statistical analysis

Statistical analysis was carried out using the Student’s *t*-test. A *P*-value <0.05 was considered statistically significantly.

## Results

### Identification of PHA genes in potato

A search in the Potato Genome Sequencing Consortium database, (http://solanaceae.plantbiology.msu.edu/pgsc_download.shtml) revealed the existence of seven sequences presenting a high degree of homology with PM H^+^-ATPases in the *S. tuberosum* Phureja genome, designated PHA1–PHA7 (Supplementary Fig. S1; Supplementary Table S2). Analysis of the protein primary structure revealed that the potato PM H^+^-ATPases present the characteristic features of PM proton pumps: the C-terminal autoinhibitory domain, 10 transmembrane segments, the small cytosolic loop, between the second and third transmembrane segments, and the large cytosolic loop, between the fourth and fifth transmembrane segments (Supplementary Fig. S1). PHA6 is truncated at its C-terminus and presents a unique region of 88 amino acids, absent in the other members of the PHA family. PHA3 also presents a 34 amino acid sequence which is missing in the other PHAs. Scanning the PM H^+^-ATPase protein sequences for motif analysis confirmed the presence of typical motifs for plant P-ATPases: TGES, crucial for ATPase activity, DKTGTLT, in which the aspartate is reversibly phosphorylated during catalysis, KGAP, essential for ATP binding, and the GDGVNDA motif, involved the hydrolysis of the acyl-phosphate intermediate (Thever and Saier, 2009). The last three motifs are not completely conserved in PHA6. The 14-3-3-binding site, located at the extreme C-terminus, is absent in PHA5 and PHA6 (Supplementary Fig. S1).

**Fig. 1.**
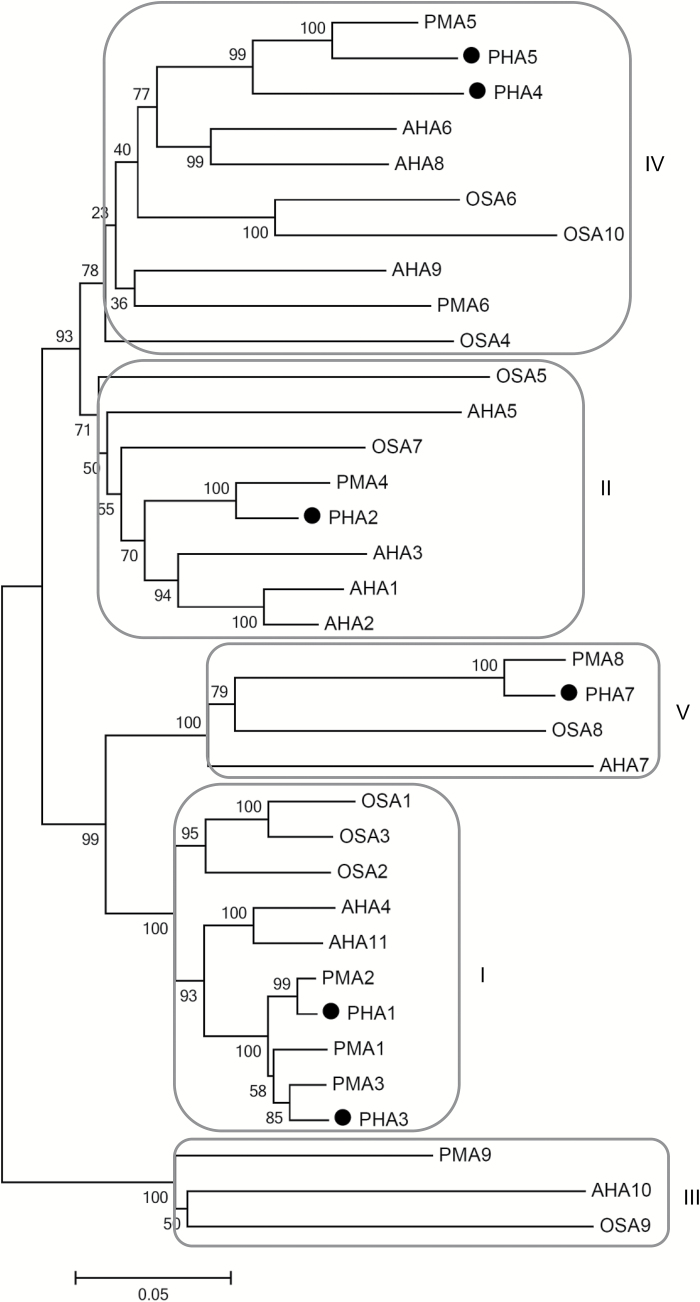
Phylogenetic analysis of potato, Arabidopsis, *N. plunbaginifolia*, and rice PM H^+^-ATPases. The amino acid sequences of PM H^+^-ATPases from *S. tuberosum* Phureja (PHA1–PHA5 and PHA7), *A. thaliana* (AHA1–AHA11), *N. plunbaginifolia* (PMA1–PMA6, PMA8, and PMA9), and *O. sativa* (OSA1–OSA10) were compared to generate the phylogenetic tree using the Neighbor–Joining method of MEGA5.05 (http://www.megasoftware.net/). The percentage of replicate trees in which the associated taxa clustered together in the bootstrap test (1000 replicates) is indicated next to the branches. GenBank accession numbers are: *A. thaliana* AHA1, P20649; AHA2, P19456; AHA3, P20431; AHA4, Q9SU58; AHA5, Q9SJB3; AHA6, Q9SH76; AHA7, Q9LY32; AHA8, Q9M2A0; AHA9, Q42556; AHA10, Q43128; AHA11, Q9LV11; *N. plumbaginifolia* PMA1, Q08435; PMA2, Q42932; PMA3, Q08436; PMA4, Q03194; PMA6, Q9SWH2; PMA8, Q9SWH1; PMA9, Q9SWH0); *O. sativa* OSA1, Q43001; OSA2, Q43002; OSA3, AF110268; OSA4, AJ440002; OSA5, AJ440216; OSA6, AJ440217; OSA7, AJ440218; OSA8, AJ440219; OSA9, AJ440220; and OSA10, AJ440221. Accession numbers for *S. tuberosum* Phureja (Potato Genome Sequencing Consortium Public Data Release): PHA1, PGSC0003DMP400055772; PHA2, PGSC0003DMP400007331; PHA3, PGSC0003DMP400043938; PHA4, PGSC0003DMP400021001; PHA5, PGSC0003DMP400013900; and PHA7, PGSC0003DMP400060260).

Multiple alignment of the protein sequences of *A. thaliana* (AHA), *N. plumbaginifolia* (PMA), *O. sativa* (OSA), and *S. tuberosum* Phureja (PHA) PM H^+^-ATPases was carried out to generate a phylogenetic tree (Fig. 1). The potato proteins were grouped into four of the five well-established subfamilies (Arango *et al.*, 2003), except for PHA6, which does not fall into any of the subfamilies defined, and was excluded from the phylogenetic analysis. PHA1–PHA5 and PHA7 were clustered into the subfamilies I, II, IV, and V, while no members of PHAs were represented in subfamily III.

### Expression profile of PHA genes

**Fig. 2.**
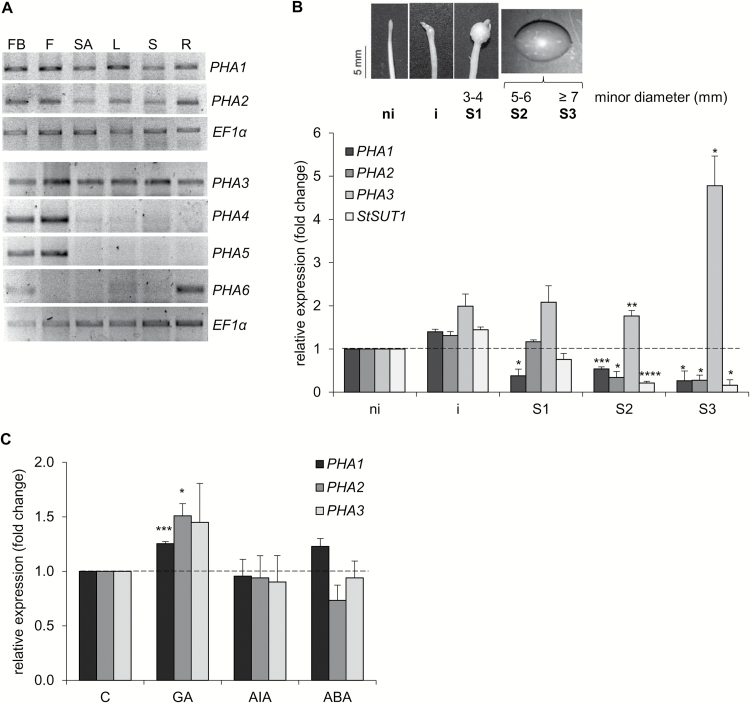
Expression profile of PHA genes. (A) Total RNA was isolated from different tissues of soil-grown potato plants, cDNA was synthesized, and semi-quantitative RT–PCR was performed. A representative RT–PCR analysis of three independent experiments is shown. FB, flower bud; F, flower; SA, shoot apex; L, leaf; S, stem; R, root. Quantitative data of RT–PCR bands are shown in Supplementary Fig. S2. (B) RT–qPCR analysis of PHA genes and *StSUT1* during tuberization *in vitro*. Total RNA was obtained from stolons cultured under tuber-inducing conditions (MS medium plus 8% sucrose) at progressive stages of tuber development (ni, non-induced stolons; i, induced stolons with visible subapical swelling; S1–S3, tubers at different stages of growth). Quantitative data (mean ±SE) of three independent experiments, each consisting of four technical replicates, are displayed in the bar graph. The asterisks indicate statistical significance (*P<0.05, **P<0.01, ***P<0.005, ****P<0.001, with respect to non-induced stolons). (C) RT–qPCR analysis of PHA genes in stolons cultured under tuber-inducing conditions for 2 weeks, in the absence or presence of 5 μM GA_3_ (GA), 25 μM 3-indoleacetic acid (IAA), or 5 μM ABA. Quantitative data (mean ±SE) of three independent experiments, each consisting of four technical replicates, are displayed in the bar graph. The asterisks indicate statistical significance (*P<0.05, ***P<0.005, with respect to control, C).

The expression profile of PHA genes in different organs was determined (Fig. 2A; Supplementary Fig. S2). *PHA1*, *PHA2*, and *PHA3* were expressed in all organs, while expression of *PHA4* and *PHA5* was restricted to flower buds and flowers. *PHA6* was expressed predominantly in flower buds and roots. *PHA7* transcripts were not detected in any of the organs analyzed (not shown).

The mRNA levels of PHA genes were determined in stolons cultured *in vitro*. *PHA1*, *PHA2*, and *PHA3* were expressed in stolons cultured under tuber-inducing conditions, while *PHA4*, *PHA5*, *PHA6*, and *PHA7* transcripts were not detected (Supplementary Fig. S3). *PHA1* and *PHA2* were expressed at higher levels in early stages of tuber organogenesis than in the tuber growth stages, while *PHA3* displayed an opposite expression pattern (Fig. 2B). The sucrose–H^+^ symporter StSUT1 showed an expression pattern similar to that of *PHA1* and *PHA2* (Fig. 2B). GA increased *PHA1* and *PHA2* expression in stolons, while no significant changes were observed for *PHA3* (Fig. 2C); no significant changes in the expression of PHA genes were detected in response to auxin or ABA (Fig. 2C). *PHA4*, *PHA5*, *PHA6*, and *PHA7* transcripts were not detected at any stage of tuberization, with or without hormone treatment (not shown).

The expression of PHA genes in response to abiotic stress was determined in leaves. Salt stress increased *PHA1*, *PHA2*, and *PHA3* mRNA levels, while drought only up-regulated the *PHA3* gene (Supplementary Fig. S4). Cold stress increased the expression of *PHA2* (Supplementary Fig. S4). *PHA4*, *PHA5*, *PHA6*, and *PHA7* transcripts were not detected in leaves in the absence or presence of stress (not shown).

### Involvement of PM H^+^-ATPase in tuberization

**Fig. 3.**
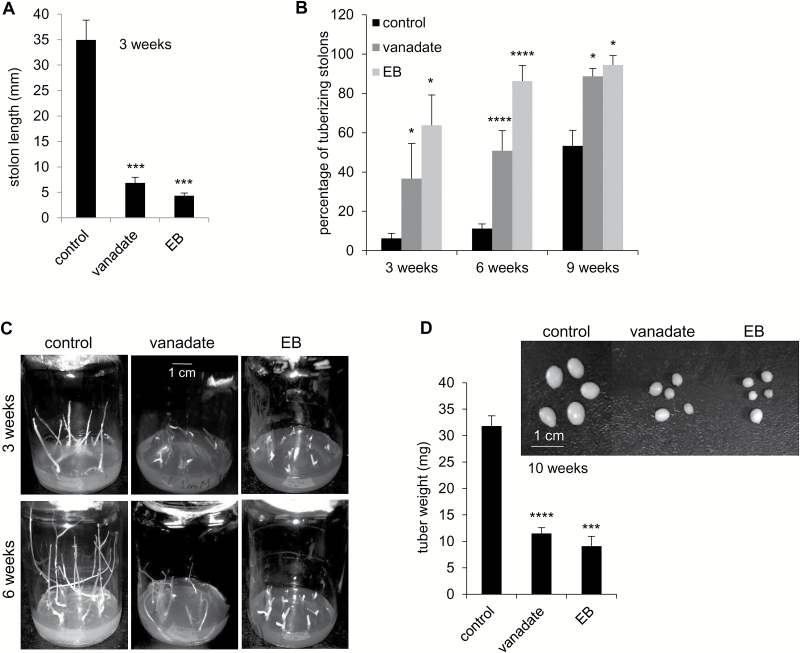
Effect of vanadate and erythrosine B (EB) on stolons cultured under tuber-inducing conditions. Stolons were cultured under tuber-inducing conditions (MS medium plus 8% sucrose) in the absence (control) or presence of 1 mM vanadate or 50 μM erythrosine B. The stolon length was measured after 3 weeks (A), and the percentage of tuberizing stolons was determined at the indicated times (B). (C) Representative images of stolons. (D) Fresh weight of tubers obtained after 10 weeks of culture under tuber-inducing conditions in the absence (control) or presence of 1 mM vanadate or 50 μM erythrosine B; a representative image of the tubers obtained is shown. Quantitative data of three independent experiments (mean ±SE) are displayed in the bar graphs. The asterisks indicate statistical significance (*P<0.05, ***P<0.005, ****P<0.001, with respect to control).

In a first attempt to elucidate the role of PHAs in tuberization, the inhibitors of H^+^-ATPase, sodium orthovanadate (vanadate) and erythrosine B (Kanczewska *et al.*, 2005; Hayashi *et al.*, 2014; Yuan *et al.*, 2017), were used in tuberization experiments *in vitro*. Dose–response experiments were carried out to determine the appropriate concentration of inhibitors, which were 1 mM and 50 μM for vanadate and erythrosine B, respectively (not shown). The length of inhibitor-treated stolons was drastically reduced (Fig. 3A, C). Both inhibitors increased the percentage of tuberizing stolons cultured under tuber-inducing conditions (MS medium plus 8% sucrose) (Fig. 3B, C). The tubers obtained from stolons treated with vanadate or erythrosine B were significantly smaller than those obtained from untreated stolons (Fig. 3D). When stolons were cultured under non-inducing conditions (MS medium plus 2% sucrose), inhibition of PM H^+^-ATPases by vanadate or erythrosine B also inhibited stolon growth and promoted tuber initiation (Supplementary Fig. S5).

### Cloning of the *PHA1* gene from *S. tuberosum* cv. Spunta

PHA1 was selected for further analysis. The full-length coding sequence of *PHA1* was cloned from *S. tuberosum* cv. Spunta. The nucleotide sequence appears in the GenBank database under the accession number KX827766. This sequence is almost identical to the *S. tuberosum* Phureja *PHA1* sequence (PGSC0003DMP400055772; Potato Genome Sequencing Consortium database), except for one nucleotide difference within the coding region, with no amino acid change. Comparing the sequence of *PHA1* from *S. tuberosum* cv. Spunta with the sequence of *PHA1* from *S. tuberosum* cv. Désirée (Harms *et al.*, 1994; GenBank accession number: X76536.1), 15 nucleotide differences were found within the coding region, one of which causes an amino acid change (R170A, in Spunta).

**Fig. 4.**
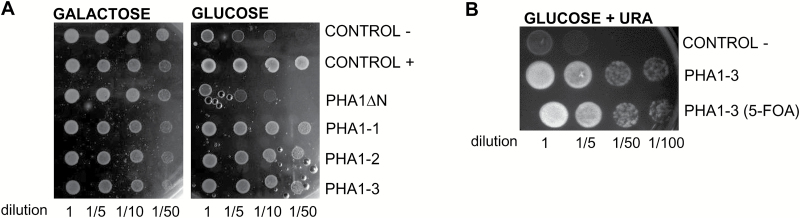
Functional complementation of a null mutation of yeast PM H^+^-ATPase (strain YAK2) by the *PHA1* gene from *S. tuberosum* cv. Spunta. (A) Serial dilutions (starting from an OD_600nm_=1.7) of the following YAK2 yeast strains were spotted onto solid media containing glucose (MGlu-His, Leu, Trp) or galactose (MGal-His, Leu, Trp), buffered at pH 6.5: Control –, YAK2 transformed with empty YEplac181; Control +, YAK2 transformed with YEplac181-E14D, that expresses the constitutively active form of the PM H^+^-ATPase PMA2 Q42932 from *N. plumbaginifolia* under the yeast *PMA1* promoter; PHA1∆N, YAK2 transformed with YEplac181-PHA1∆N that expresses a truncated, inactive form of PHA1, lacking the first 553 nucleotides of the N-terminus (devoid of the TGES motif) under the yeast *PMA1* promoter; PHA1-1/2/3, YAK2 transformed with YEplac181-PHA1, that expresses *PHA1* under the yeast *PMA1* promoter (three different transformed yeast clones were used). (B) Serial dilutions (starting from an OD_600nm_=1.7) of the following YAK2 yeast strains spotted onto solid media containing glucose (MGlu-His, Leu, Ura, Trp): Control –, PHA1–PHA3, and PHA1–PHA3 after 5-FOA treatment to eliminate the plasmid bearing the yeast PM H^+^-ATPase gene. Yeast strains were grown at 30 °C for 48 h.

To confirm that the *PHA1* gene cloned encodes a protein with PM H^+^-ATPase activity, a functional complementation assay was carried out. The *S. tuberosum* cv. Spunta *PHA1* coding sequence was subcloned in YEplac181 under the control of the constitutive promoter of the yeast *PMA1* gene. This plasmid was introduced into the yeast strain YAK2, which is deleted from its own two PM H^+^-ATPase genes (*PMA1* and *PMA2*), and is able to survive on galactose medium with a centromeric plasmid carrying the yeast *PMA1* controlled by the *GAL1* promoter (de Kerchove d’Exaerde *et al.*, 1995). Three independent transformants (PHA1-1, PHA1-2, and PHA1-3) obtained in a galactose medium were able to sustain the growth of YAK2 yeast cells when shifted to a glucose medium (Fig. 4A), demonstrating that the *PHA1* gene cloned from *S. tuberosum* cv. Spunta has ATPase and proton pumping activity. The same result was obtained after eliminating the plasmid bearing the yeast PM H^+^-ATPase from the YAK2-PHA1-3 strain by 5-FOA (Fig. 4B).

### Overexpression of *PHA1* promotes stolon growth and branching, and increases tuber weight

**Fig. 5.**
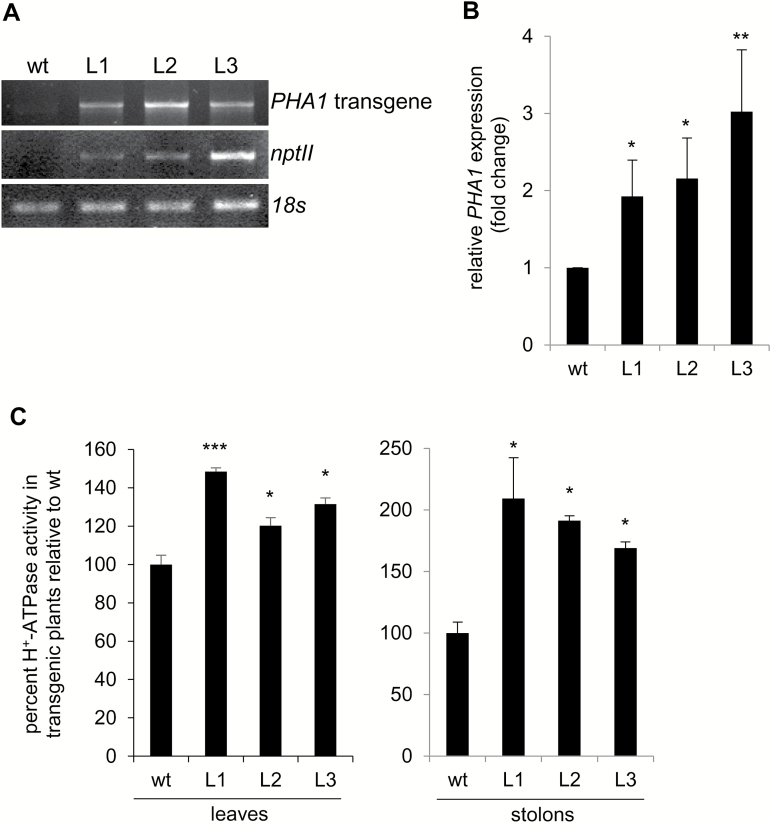
Analysis of transgenic plants overexpressing *PHA1* (PHA1-OE). (A) PCR analysis of genomic DNA isolated from leaves of wild-type (wt) and transgenic plants (L) grown *in vitro*, to detect the presence of the transgene and the *nptII* gene (B) RT–qPCR analysis of RNA isolated from leaves of wild-type and transgenic plants grown *in vitro*, to determine *PHA1* expression. Data are presented as the expression level relative to the wt. Quantitative data (mean ±SE) of three independent experiments, each consisting of four technical replicates, are displayed in the bar graph. (C) PM H^+^-ATPase activity in leaves and stolons, expressed as the percentage increase in proton pump activity of transgenic lines with respect to wild-type plants. As a reference, average PM H^+^-ATPase activity was 44.7 pmol Pi min^–1^ µg^–1^ protein for wild-type leaves and 18.7 pmol Pi min^–1^ µg^–1^ protein for wild-type stolons. Means ±SE of two independent experiments each performed in quadruplicate, are shown. The asterisks indicate statistical significance (*P<0.05, **P<0.01, ***P<0.005, with respect to the wt).

Transgenic plants expressing the *PHA1* gene under the control of the 35S CaMV promoter (PHA1-OE) were developed. Three independent PHA1-OE transgenic lines (L1–L3) were selected for detailed characterization. The presence of the transgene was confirmed by PCR amplification (Fig. 5A). RT–qPCR analysis showed that the mRNA abundance of *PHA1* was higher in transgenic lines than in wild-type plants (Fig. 5B). Accordingly, transgenic lines exhibited a higher PM H^+^-ATPase activity in leaves and stolons (Fig. 5C).

**Fig. 6.**
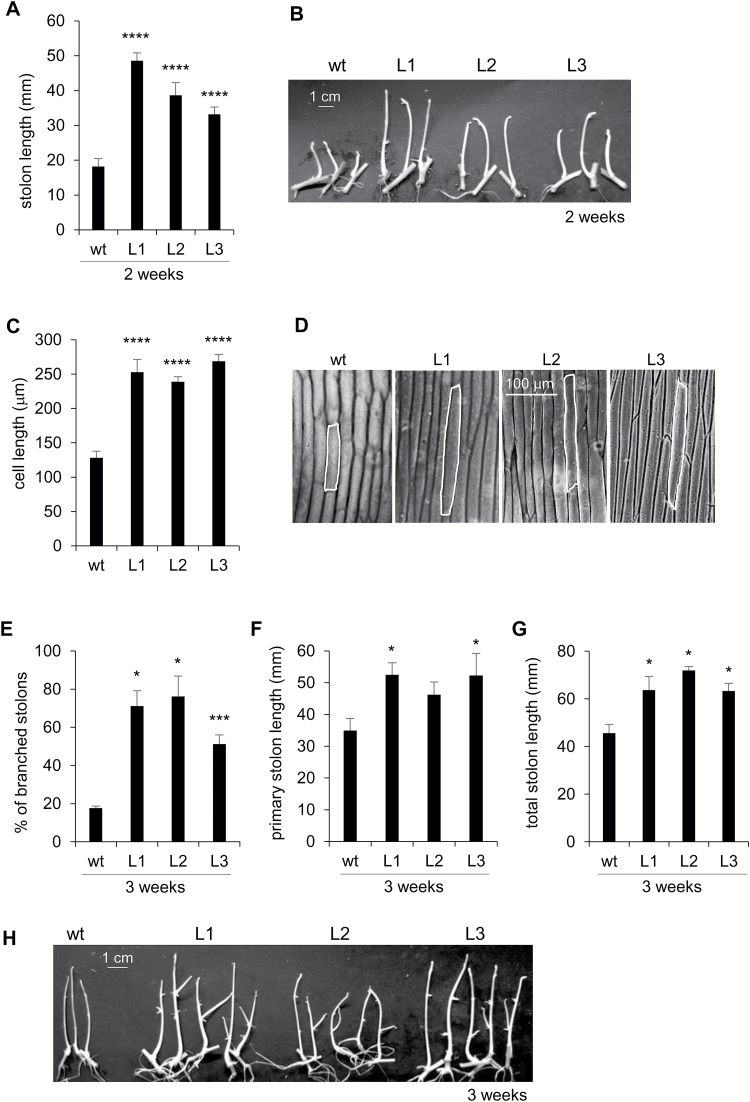
Phenotypic analysis of PHA1-OE stolons cultured *in vitro*. Stolons from wild-type (wt) and PHA1-OE plants (L) were cultured under tuber-inducing conditions (MS medium plus 8% sucrose). (A) Stolon length was determined after 2 weeks of culture; quantitative data of three independent experiments (mean ±SE) are displayed in the bar graph; a representative image is shown (B). (C) Longitudinal length of epidermal cells of the medial region of the stolons cultured for 2 weeks; the data represent the means ±SE of five biological replicates; lengths of 30–60 cells per replicate were measured. (D) Imprints of epidermal cells from the medial region of stolons, viewed by light microscopy. After 3 weeks, the percentage of branched stolons (E), primary stolon length (F), and total stolon length (G) were determined; quantitative data of three independent experiments (mean ±SE) are displayed in the bar graphs. (H) Representative images of stolons after 3 weeks of culture. The asterisks indicate statistical significance (*P<0.05, ***P<0.005, ****P<0.001, with respect to the wt).

**Fig. 7.**
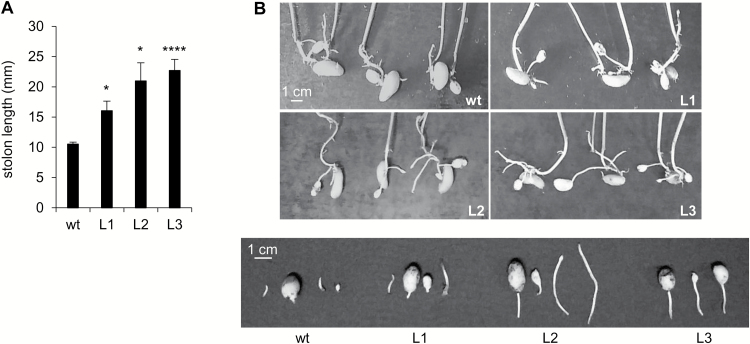
Stolon length of PHA1-OE plants grown in soil. (A) Stolon length of plants obtained from seed tubers, grown in soil, in a greenhouse, for 4 weeks. The data of four independent experiments (mean ±SE) are displayed in the bar graph; each experiment consisted of 3–4 soil-grown plants per condition, with 3–5 stolons per plant. The asterisks indicate statistical significance [*P<0.05, ****P<0.001, with respect to the wild type (wt)]. (B) Representative image of the underground part of the plants (upper panel) and detached stolons (lower panel).

After 2 weeks of culture under tuber-inducing conditions, PHA1-OE stolons were longer than wild-type stolons (Fig. 6A, B). Accordingly, transgenic stolons had longer epidermal cells (Fig. 6C, D). No branching was observed after 2 weeks (Fig. 6B). After 3 weeks of culture, the percentage of branched stolons was significantly higher in PHA1-OE stolons than in wild-type stolons (Fig. 6E, H). The primary stolon length of L1 and L3 was higher, with no statistically significant difference between L2 and wild-type stolons (Fig. 6F), although the total stolon length was increased in all three transgenic lines, due to the increased number of branches (Fig. 6G). PHA1-OE plants grown in soil presented longer stolons than wild-type plants (Fig. 7A, B), confirming the long-stolon phenotype observed *in vitro*.

**Fig. 8.**
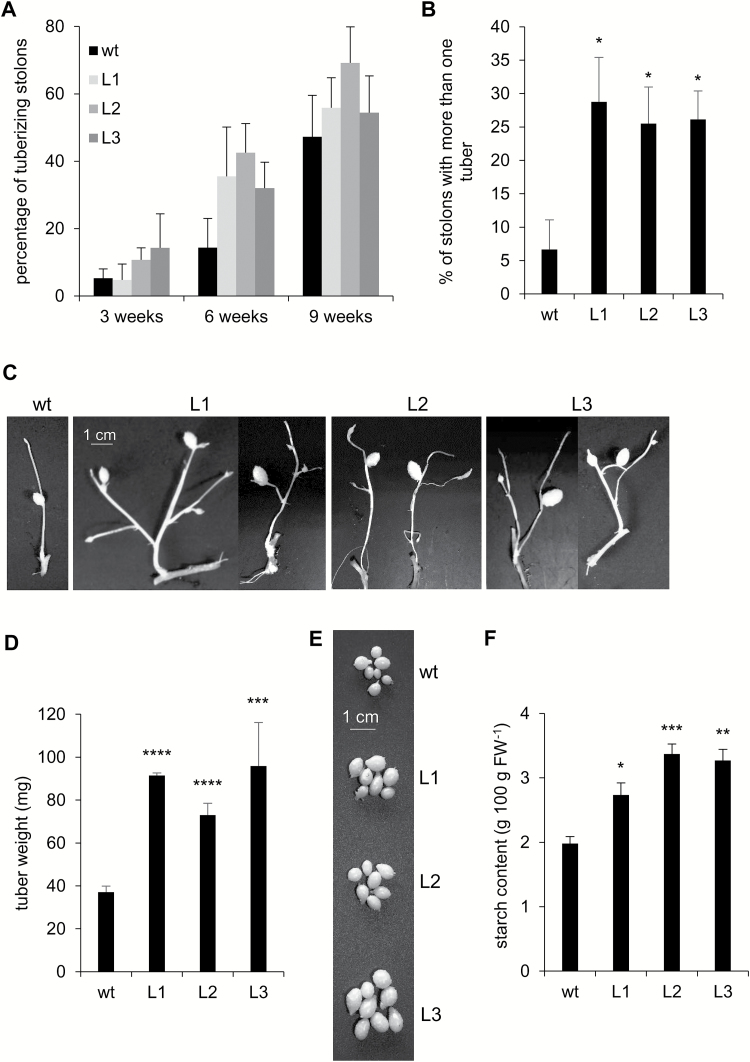
Tuberization of PHA1-OE stolons cultured *in vitro*. Stolons from wild-type (wt) and PHA1-OE (L) plants were cultured under tuber-inducing conditions (MS medium plus 8% sucrose). (A) Percentage of tuberizing stolons, determined at the indicated times. (B) Percentage of stolons presenting more than one tuber after 10 weeks of culture; a representative image of the stolons is shown (C). (D) Fresh weight of tubers obtained from wild-type and PHA1-OE stolons after 10 weeks of culture; a representative image of the tubers is shown (E). (F) Starch content of tubers obtained from wild-type and PHA1-OE stolons after 10 weeks of culture. Quantitative data of three independent experiments (mean ±SE) are displayed in the bar graphs. The asterisks indicate statistical significance (*P<0.05, **P<0.01, ***P<0.005, ****P<0.001, with respect to the wt).

The percentage of tuberizing stolons was not significantly affected in PHA1-OE lines (Fig. 8A); however, after 10 weeks of culture, ~26% of PHA1-OE stolons presented more than one tuber (2–5 tubers per stolon), while only 7% of wild-type stolons developed more than one tuber (Fig 8B, C). In most cases, the additional tubers were very small or undeveloped. The tubers developed from PHA1-OE stolons were larger and presented higher starch content than those obtained from wild-type stolons (Fig. 8D–F).

**Fig. 9.**
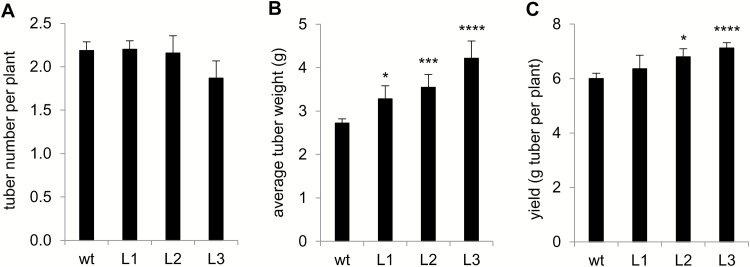
Tuberization of PHA1-OE plants in soil. Wild-type (wt) and PHA1-OE plants (L) transferred to soil *ex vitro* were cultivated in a growth chamber. After 10 weeks, the tubers were harvested and the number of tubers obtained per plant (A), the average tuber fresh weight (B), and the tuber yield, defined as grams (FW) of tuber obtained per plant (C) were determined. Data are the results (mean ±SE) of 30–60 plants obtained from five different harvests performed over an 18 month period. The asterisks indicate statistical significance (*P<0.05, ***P<0.005, ****P<0.001, with respect to the wt).

The tuberization capacity of PHA1-OE plants grown in soil was determined. There were no significant differences in the number of tubers obtained per plant between PHA1-OE and the wild type (Fig. 9A). The average weight of tubers obtained from PHA1-OE plants was higher than that of wild-type tubers (Fig. 9B). The tuber yield per plant was higher in transgenic lines with respect to wild-type plants, although this difference was statistically significant only for L2 and L3 (Fig. 9C). It is important to note that the tuber yield was determined in plants transferred to soil *ex vitro*, which are much smaller than potato plants obtained from seed tubers, and consequently have significantly lower yields.

### Overexpression of PHA1 alters cell wall invertase activity, starch content, and *StSUT1* expression in stolons

**Fig. 10.**
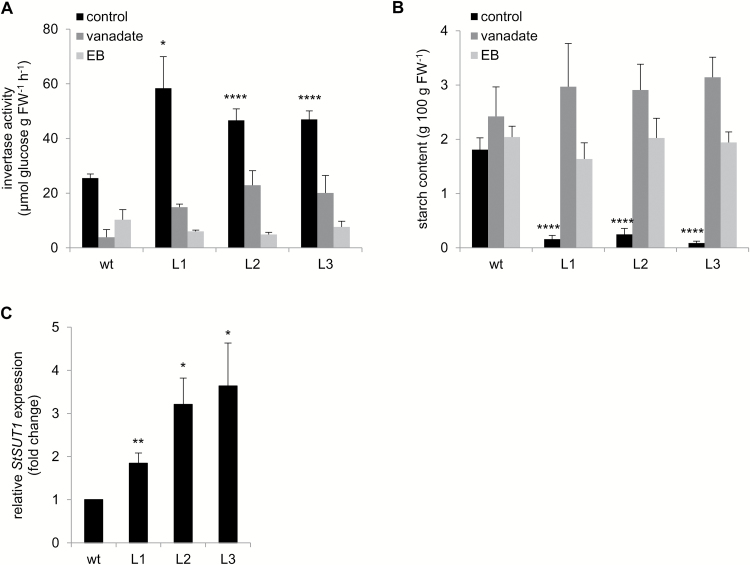
Cell wall acid invertase activity, starch content, and *StSUT1* expression in stolons from wild-type (wt) and PHA1-OE (L) plants. Stolons were cultured under tuber-inducing conditions (MS medium plus 8% sucrose) for 2 weeks; alternatively, 1 mM vanadate or 50 μM erythrosine B (EB) was applied to the medium. (A) Cell wall acid invertase activity was determined in the apical portion (1 cm) of stolons cultured in the absence or presence of PM H^+^-ATPase inhibitors. (B) Starch content was determined in the apical portion (1 cm) of stolons cultured in the absence or presence of PM H^+^-ATPase inhibitors. (C) RT–qPCR analysis of *StSUT1* in wt and transgenic stolons; data are presented as the expression level relative to the wt. Quantitative data (mean ±SE) of three independent experiments, each consisting of four technical replicates, are displayed in the bar graphs. The asterisks indicate statistical significance (*P<0.05, **P<0.01, ****P<0.001, with respect to the wt).

Cell wall invertase activity, starch content, and *StSUT1* expression were determined in stolons during the elongation stage. After 2 weeks of culture, PHA1-OE stolons showed higher cell wall invertase activity (Fig. 10A) and accumulated significantly less starch than wild-type stolons, showing very low values of starch content (Fig. 10B). Treatment with H^+^-ATPase inhibitors inhibited stolon elongation (Supplementary Fig. S6), decreased cell wall invertase activity (Fig. 10A), and increased starch accumulation (Fig. 10B) in PHA1-OE stolons, abolishing the phenotypic differences between transgenic and wild-type stolons. PHA-OE stolons showed higher expression levels of the sucrose–H^+^ symporter gene *StSUT1* than wild-type stolons (Fig. 10C).

### Overexpression of PHA1 promotes stem growth and branching

**Fig. 11.**
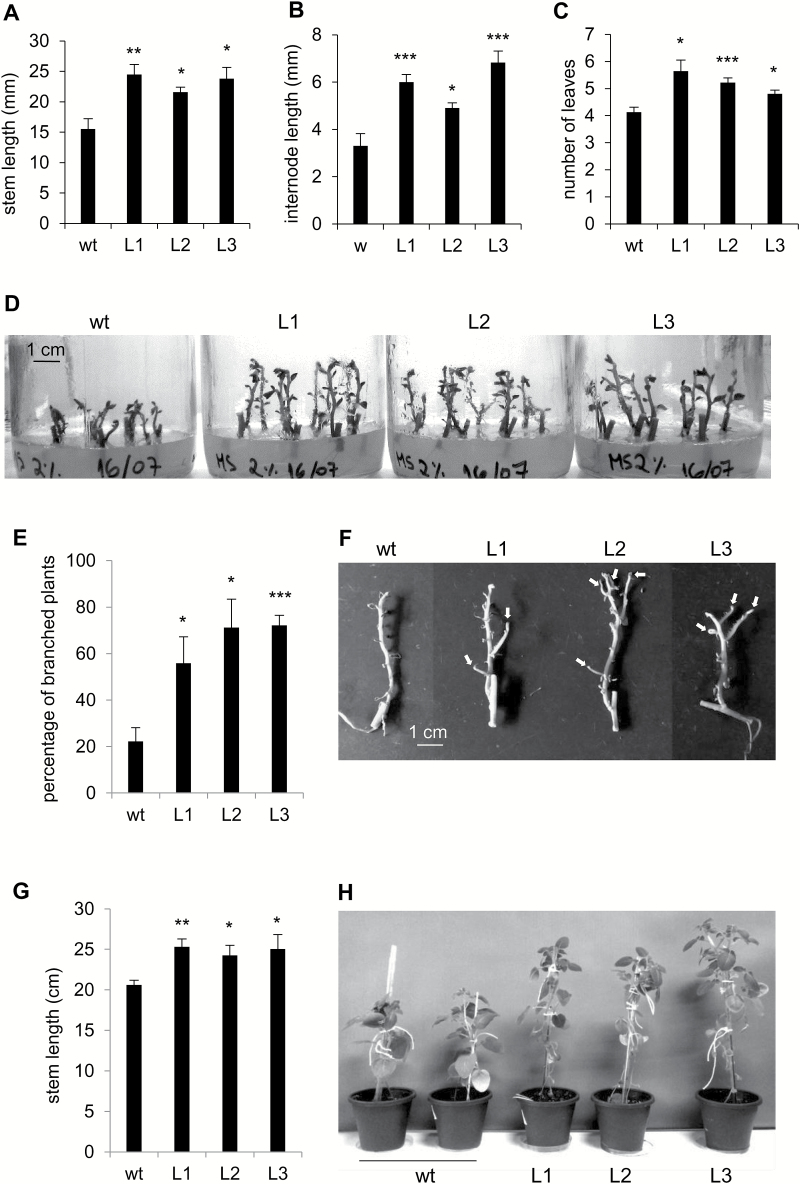
Phenotypic analysis of PHA1-OE plants grown *in vitro*. Wild-type (wt) and transgenic (L) plants generated from single-node cuttings were grown on MS medium plus 2% sucrose. After 2 weeks, the stem length (A), first internode length (B), and number of leaves (C) were determined. (D) Representative image of plants after 2 weeks of culture. (E) After 4 weeks, the percentage of branched plants was determined as the number of plants presenting at least one branch ≥5 mm, with respect to the total number of plants. (F) Representative image of wt and PHA1-OE plants grown *in vitro* for 4 weeks; leaves appear shrunken, since plants were allowed to air-dry for better visualization of branches, which are indicated with arrows. (G) Stem length of plants grown in soil, in a growth chamber, for 4 weeks after *ex vitro* transfer; a representative image of the plants is shown (H). Quantitative data of four independent experiments (mean ±SE) are displayed in the bar graphs; each experiment consisted of 15–20 *in vitro* cultured plants or 8–10 soil-grown plants per condition. The asterisks indicate statistical significance (*P<0.05, **P<0.01, ***P<0.005, with respect to the wt).

Plant growth parameters were determined in 2-week-old plants generated *in vitro* from internode cuttings. PHA1-OE plants showed higher stem and first internode lengths, and higher number of leaves than wild-type plants (Fig.11A–D). No significant differences were observed in the number of primary roots or total root length, between transgenic and wild-type plants (Supplementary Fig. S7). Inhibition of PM H^+^-ATPase activity by vanadate or erythrosine B resulted in shorter plants (Supplementary Fig. S8). No branching was observed in 2-week-old plants (Fig. 11D). After 4 weeks of growth *in vitro*, the percentage of plants presenting branches was significantly higher in PHA1-OE plants than in wild-type plants (Fig. 11E, F); a similar result was obtained using plants generated from shoot apexes (Supplementary Fig. S9). PHA1-OE plants grown in soil were ~20% taller than wild-type plants (Fig. 11G, H).

**Fig. 12.**
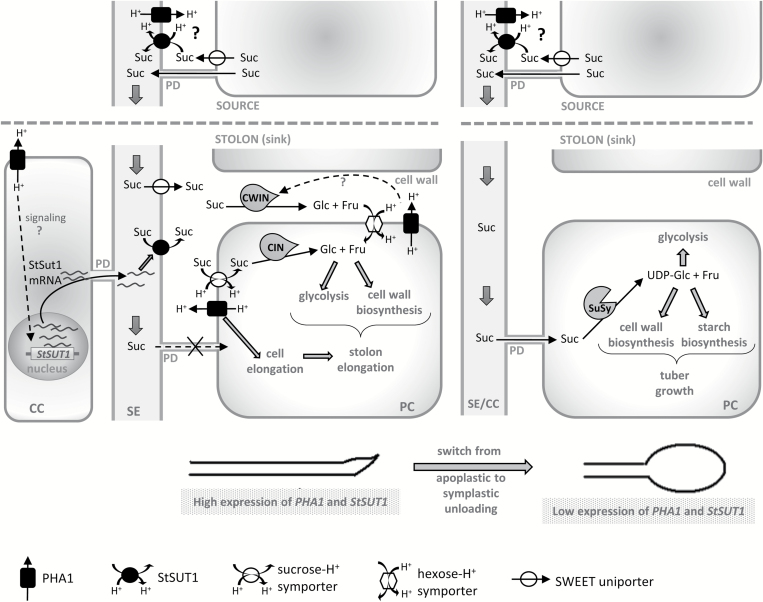
Model for the potential function of PHA1 in stolons, based on the results obtained in this study (see text for details). Suc, sucrose; SE, sieve element; CC, companion cell; CWIN, cell wall invertase; CIN, cytoplasmic invertase; SuSy, sucrose synthase; PD, plasmodesmata; PC, parenchymal cell.

## Discussion

### The potato PM H^+^-ATPase family

An *in silico* screening in the *S. tuberosum* Phureja genome database retrieved seven sequences, designated PHA1–PHA7, with a high degree of homology with PM H^+^-ATPases (Fig. 1; Supplementary Table S2; Supplementary Fig. S1), including two genes (*PHA1* and *PHA2*) identified earlier in *S. tuberosum* cv. Désirée (Harms *et al.*, 1994).

The expression patterns of PHAs were determined in *S. tuberosum* cv. Spunta. *PHA1*, *PHA2*, and *PHA3* are ubiquitously expressed throughout the plant (Fig. 2A). These results are consistent with previous reports describing the expression patterns in other species. PHA1 and PHA3 belong to subfamily I, and PHA2 to subfamily II; the genes of these subfamilies (*A. thaliana AHA1*, *AHA2*, *AHA3*, *AHA4*, and *AHA11*; tobacco *PMA1*, *PMA2*, *PMA3*, and *PMA4*; maize *MHA2*; tomato *LHA1*; and cucumber *CsHA2*, *CsHA3*, *CsHA4*, *CsHA8*, *CsHA9*, and *CsHA10*) have shown a broad expression pattern throughout the plant (Ewing and Bennett, 1994; Frías *et al.*, 1996; Moriau *et al.*, 1999; Oufattole *et al.*, 2000; Santi *et al.*, 2003; Lefebvre *et al.*, 2005; Gaxiola *et al.*, 2007; Wdowikowska and Klobus, 2016). In contrast, *PHA4* and *PHA5* expression is limited to flower buds and flowers, while *PHA6* is only expressed in flower buds and roots (Fig. 2A), suggesting that the pumps encoded by these genes have more specialized functions. PHA4 and PHA5 belong to subfamily IV; it has been shown that the expression of genes of this subfamily is limited to floral organs: Arabidopsis *AHA6* and *AHA9* transcripts are present only in anthers, *AHA8* and the tobacco *PMA5* are expressed only in pollen, and cucumber *CsHA5*, *CsHA6*, and *CsHA7* are exclusively expressed in young flowers (Houlne and Boutry, 1994; Lefebvre *et al.*, 2005; Gaxiola *et al.*, 2007; Wdowikowska and Klobus, 2016). Salt stress significantly increases *PHA1*, *PHA2*, and *PHA3* expression (Supplementary Fig. S4), suggesting a role for these PM H^+^-ATPases in salinity tolerance, as described in previous reports (Harms *et al.*, 1994; Kalampanayil and Wimmers, 2001; Janicka-Russak and Klobus, 2007; Sahu and Shaw, 2009).

Only *PHA1*, *PHA2*, and *PHA3* are expressed in stolons (Supplementary Fig. S3), and their expression levels change during the tuberization process (Fig. 2B), suggesting a role for these PM H^+^-ATPases in the development of stolons and tubers. Inhibition of PM H^+^-ATPase activity by vanadate or erythrosine B inhibits stolon elongation, promotes tuber induction, and impairs tuber growth (Fig. 3; Supplementary Fig. S5), confirming that PM H^+^-ATPases are involved in tuberization acting at different stages of the process.

### PHA1 promotes stolon elongation

PHA1 was selected for functional analysis by overexpression in potato plants. PHA1-OE stolons elongate faster and present longer epidermal cell length than wild-type stolons after 2 weeks of culture (Fig. 6A–D), indicating that PHA1 activity contributes to the stolon elongation that occurs prior to tuber initiation by promoting cell elongation. In agreement with this, inhibition of PM H^+^-ATPase activity with vanadate or erythrosine B results in shorter stolon length (Fig. 3A; Supplementary Fig. S5A). These results are consistent with the role of PM H^+^-ATPases in cell elongation and expansion described for other tissues such as hypocotyls and roots (Haruta and Sussman, 2012; Inoue *et al.*, 2016), and *Nicotiana tabacum* BY-2 cells (Niczyj *et al.*, 2016). Acidification of the apoplasm by PM H^+^-ATPases leads to the wall-loosening process and cell elongation (reviewed in Hager, 2003). GA promotes stolon elongation by stimulating both cell division and cell elongation (Loy, 1977; Xu *et al.*, 1998). *PHA1* expression is induced in stolons in response to GA (Fig. 2C), thus it is possible to speculate that PHA1 mediates the GA-induced stolon elongation. However, more studies are needed to establish the mechanistic link between GA and PHA1.

### PHA1 regulates sucrose–starch metabolism in stolons

During the stolon elongation phase, apoplastic sucrose unloading predominates (Viola *et al.*, 2001). Although the exact mechanism occurring during this process remains unknown, sucrose can be unloaded from the phloem into the apoplast by the sucrose–H^+^ symporter StSUT1 in its inverse transport mode (Kühn *et al.*, 2003; Carpaneto *et al.*, 2005), or possibly by recently discovered facilitators of the SWEET family (Chen *et al.*, 2012). The apoplastic sucrose can be converted to hexoses by a cell wall invertase, and taken up by the parenchyma cells through hexose–H^+^ symporters, not yet characterized in potato. Sucrose uptake from the aploplast might be mediated by sucrose–H^+^ symporters, possibly different from StSUT1, since it was reported that StSUT1 is localized in sieve elements but not in storage parenchyma in sink tubers (Kühn *et al.*, 2003) (Fig. 12). Apoplastic unloading correlates with high acid invertase activity (Ross *et al.*, 1994; Appeldoorn *et al.*, 1997; Viola *et al.*, 2001), and is associated with rapidly growing vegetative sink tissues (Ehness and Roitsch, 1997; Herbers and Sonnewald, 1998). During the transition from stolon to tuber, a switch from apoplastic to symplastic unloading occurs, accompanied by a switch from an invertase–sucrolytic to a sucrose synthase (SuSy)–sucrolytic pathway, leading to the starch accumulation phase and tuber growth (Viola *et al.*, 2001) (Fig. 12).

During the elongation phase, PHA1-OE stolons grow faster (Fig. 6A), and present higher levels of cell wall invertase activity (Fig. 10A) and significantly lower starch content than wild-type stolons; moreover, starch is almost undetectable in transgenic stolons (Fig. 10B). These metabolic differences are accompanied by higher expression levels of *StSUT1* in PHA1-OE stolons (Fig. 10C). In agreement with this, the PHA1-OE stolon phenotype can be reversed by the application of PM H^+^-ATPase inhibitors, that results in shorter length, decreased cell wall invertase activity, and increased starch content (Fig. 10A, B; Supplementary Fig. S6). Based on these data, it may be hypothesized that, besides promoting stolon growth by mediating cell elongation, PHA1 regulates the sucrose–starch metabolism in elongating stolons (Fig. 12). PHA1 positively regulates cell wall invertase activity (by an unknown mechanism), and its proton pumping activity can sustain the secondary transport of hexose–H^+^ and sucrose–H^+^ symporters, promoting the uptake of hexoses and sucrose by parenchyma cells; moreover, PHA1 increases the expression of *StSUT1* in stolons, promoting sucrose unloading into the apoplast (Fig. 12). By doing so, PHA1 favors apoplastic unloading, sucrolysis by invertase and stolon elongation, and prevents starch accumulation, which is associated with symplastic unloading. Supporting this hypothesis, *PHA1* and *StSUT1* expression is high in wild-type stolons during the elongation phase, and decreases during tuber growth (Fig. 2B). In this context, PHA1 seems to play a key role in the molecular mechanism that determines the switch from the apoplastic unloading/invertase–sucrolytic pathway to the symplastic unloading/SuSy–sucrolytic pathway.

PHA1-OE stolons show higher expression levels of *StSUT1* (Fig. 10C), suggesting that this PM H^+^-ATPase may be part of the signaling pathway that leads to the activation of *StSUT1* transcription. It is possible that PHA1 induces the expression of *StSUT1* in companion cells, and the StSUT1 mRNA is transported via plasmodesmata to the sieve elements as previously described (Kühn *et al.*, 1997) (Fig. 12).

### Role of PHA1 in tuber development

PHA1-OE tubers are larger and present higher starch content than wild-type tubers (Figs 8D–F, 9B). StSUT1 is essential for sucrose phloem loading (Kühn *et al.*, 1996); therefore, it is possible to speculate that PHA1 might enhance sucrose phloem loading by energizing this sucrose–H^+^ symporter (Fig. 12), promoting sucrose translocation to sink tubers and starch accumulation. The mechanism of sucrose phloem loading in whole plants is different from the process occurring in stolons cultured *in vitro*; however, in both cases sucrose loading requires a proton symport mechanism energized by PM H^+^-ATPases. In plants, sucrose is loaded into the phloem in source leaves (Lemoine *et al.*, 2013), while in stolons cultured *in vitro*, sucrose is obtained from the culture medium and loaded into the phloem as either hexoses (after hydrolysis by invertase) or sucrose (De Riek *et al.* 1997).

PHA1-OE stolons show an increased branching phenotype (Fig. 6E, H), which results in a higher percentage of stolons presenting more than one tuber, although the additional tubers are undeveloped (Fig. 8B, C). Stolon branching increases the number of potential tuber sites; however, developing more than one tuber per stolon leads to a competition for resources that negatively affects tuber size (Celis-Gamboa *et al.*, 2003). Interestingly, the fully developed tubers obtained from PHA1-OE stolons are larger than those from wild-type stolons (Fig. 8D, E), indicating that PHA1 positively regulates tuber growth. This result agrees with the observation that inhibition of PM H^+^-ATPase activity results in smaller average tuber weight (Fig. 3D). There are no significant differences in the percentage of tuberizing stolons between PHA1-OE and wild-type plants (Fig. 8A); however, treatment with vanadate or erythrosine B enhances tuber induction (Fig. 3B; Supplementary Fig. S5B); this effect might be due to the inhibition of PHA2 or PHA3, which are also expressed in stolons, or to a non-specific action.

As observed *in vitro*, PHA1-OE plants grown in soil also develop larger tubers and show an increased tuber yield with respect to wild-type plants (Fig. 9B, C), suggesting that the *PHA1* gene might be a potential tool to increase potato crop yield.

### Branching phenotype of PHA1-OE plants

Stems of PHA1-OE plants grown *in vitro* present a highly branched phenotype, similar to PHA1-OE stolons (Fig. 11E, F; Supplementary Fig. S9). It has recently been shown that lateral bud growth depends on the amount of sucrose translocated to those buds, with sugar distribution, not auxin, being the initial regulator of apical dominance (Mason *et al.*, 2014). It is now clear that sugar supply to axillary buds is not only essential to trigger outgrowth, but is also required to release bud dormancy (Barbier *et al.*, 2015). Sucrose transporters and PM H^+^-ATPases have been implicated in this process; in rose bush, the onset of bud outgrowth correlates with increased sugar translocation to axillary buds and the up-regulation of the sucrose–H^+^ symporter gene *RhSUC2* (Girault *et al.*, 2010; Henry *et al.*, 2011); the dormancy break of *Prunus persica* buds is associated with sucrose uptake and PM H^+^-ATPase activity (Aue *et al.*, 1999; Maurel *et al.*, 2004). The branched phenotype of PHA1-OE plants could be due to an increased sucrose unloading towards the lateral buds via sucrose–H^+^ symporters energized by PHA1; however, more studies are required to determine the role of PHA1 in axillary bud outgrowth.

### PHA1 promotes plant growth

Like PHA1-OE stolons, the stems of PHA1-OE plants grown *in vitro* elongate faster, and show longer internodes and more leaves than wild-type plants (Fig. 11A–D). Likewise, PHA1-OE plants grown in soil are taller than wild-type plants (Fig. 11G, H). Thus, PHA1 mediates growth in the aerial part of the plant as well as in underground stolons. Accordingly, inhibition of PM H^+^-ATPase activity leads to a reduction in stem length (Supplementary Fig. S8). There is much evidence for the role of PM H^+^-ATPase in plant growth based on studies using proton pump inhibitors; however, strong genetic evidence has so far been sparse. It was reported that overexpression of the unmodified PM H^+^-ATPase gene *PMA4* in tobacco has no effect on plant growth; however, the constitutive expression of a constitutively activated form of PMA4 results in abnormal leaf inclination and twisted stems, suggesting alterations in cell expansion (Gévaudant *et al.*, 2007). Another study has shown that the Arabidopsis *aha2* mutant completes its life cycle without any observable growth alteration, and the reduced-growth phenotype only becomes apparent under stress conditions that reduce the transmembrane electrical gradient and/or external proton chemical gradient (Haruta and Sussman, 2012). The present study provides strong genetic evidence for the role of PHA1 as a driver of growth in potato plants, showing a clear enhanced-growth phenotype of PHA1-OE plants.

## Supplementary data

Supplementary data are available at *JXB* online.

Table S1. List of primers used for semi-quantitative RT–PCR.

Table S2. Chromosome localization, coding region length, predicted protein length, and molecular weight of the PHA isoforms.

Fig. S1. Alignment of the protein sequences of PHA isoforms.

Fig. S2. Quantification of RT–PCR bands of Fig. 2A.

Fig. S3. RT–PCR analysis of PHA genes in stolons.

Fig. S4. Expression of PHA genes in response to abiotic stress.

Fig. S5. Effect of vanadate and erythrosine B on stolons cultured under non-inducing conditions.

Fig. S6. Stolon length of PHA1-OE plants cultured in the presence of PM H^+^-ATPase inhibitors.

Fig. S7. Phenotypic analysis of PHA1-OE plants.

Fig. S8. Effect of vanadate and erythrosine B on stem length.

Fig. S9. Branching phenotype of PHA1-OE plants.

## Supplementary Material

Supplementary Tables S1-S2 Figures S1-S9

## Abbreviations:

EB: erythrosine B
GA: gibberellic acid.
