# Supplementary material for: The plasma membrane H+-ATPase gene family in Solanum tuberosum L. Role of PHA1 in tuberization
Source: J Exp Bot. 2017 Aug 19;68(17):4821–37. doi: 10.1093/jxb/erx284 (PMC5853856; doi:10.1093/jxb/erx284)
Supplement: Supplementary Tables S1-S2 Figures S1-S9 [file erx284_suppl_supplementary_tables_s1_s2_figures_s1_s9.pdf]

Table S1. Primers and reaction conditions used for the determination of PHA isoforms expression by semi-quantitative RT-PCR.

	Primer sequence	Annealing temperature (°C)	Number of cycles
PHA1 forward	5’ GTGAGTGGGTGATAGAATGAAGAC 3’	46	20
PHA1 reverse	5’ GCCGATAATGAATGCTGTTATAG 3’	46	20
PHA2 forward	5’ AGAAAAGAAGAGACACACAAGC 3’	46	24
PHA2 reverse	5’ GACACAATCCCTTTCAATGG 3’	46	24
PHA3 forward	5' TGATCAAGTTCCTCATCCG 3'	47	26
PHA3 reverse	5' TTAAACGGTGTATGACTGCTG 3'	47	26
PHA4 forward	5' GACACAATGATCCAAAACAAGAC 3'	48	30
PHA4 reverse	5' ATTCAACAAAATGCCAGCAG 3'	48	30
PHA5 forward	5' GTG ATGCCTGGGATTCAATG 3'	48	30
PHA 5 reverse	5' CTCCCATAATTCATTGCATAATCTTAG 3'	48	30
PHA6 forward	5' CCTGGTTGTGATAGTCTCC 3'	47	35
PHA6 reverse	5' AATCACAATATGCTTTTCAAAAATCC 3'	47	35
PHA7 forward	5' GATCCCATCAAATTTGCTG 3'	46	30
PHA7 reverse	5' GGTGTAATGCGGATTGAC 3'	46	30
EF1-α forward	5´ GTATGGTTGTGACCTTTGG 3´	48	20
EF1-α reverse	5´ CAACATTCTTGACAACAC 3´	48	20

Table S2. Chromosome localization, coding region length, predicted protein length and molecular weight (MW) of the plasma membrane H^+-ATPases identified in *S. tuberosum* Phureja (Potato Genome Sequencing Consortium database).

name	transcript ID	peptide ID	chromosome localization	coding region length (bp)	protein length (aa)	protein MW (KDa)
**PHA1**	PGSC0003DM T400083041	PGSC0003DM P400055772	6	2871	956	105.0
**PHA2**	PGSC0003DM T400010497	PGSC0003DM P400007331	7	2859	952	105.0
**PHA3**	PGSC0003DM T400065168	PGSC0003DM P400043938	3	2973	990	109.4
**PHA4**	PGSC0003DM T400030913	PGSC0003DM P400021001	7	2856	951	104.5
**PHA5**	PGSC0003DM T400020414	PGSC0003DM P400013900	12	2775	924	101.6
**PHA6**	PGSC0003DM T400003602	PGSC0003DM P400002571	2	2739	912	101.7
**PHA7**	PGSC0003DM T400088585	PGSC0003DM P400060260	8	2901	966	106.2

```
PHA6      -----------MGLDDLVDLENIPIEQVFENLKCNKEGLTYTDVEERLSIYGHNKLEEKT    49
PHA7      MGTEKTTTTLEAINNETVDLENIPIKEVFENLKCTEEGLNSAEVEKRLNVFGHNKLEEKK    60
PHA1      --MGEKPEVLDAVLKETVDLENIPIEEVFENLRCTKEGLTGTAAQERLAIFGYNKLEEKK    58
PHA3      --MAEKPEVLDAVLKETVDLENIPIEEVFENLRCTREGLTTTAAQERLSIFGYNKLEEKK    58
PHA2      ---MAKAISLEEIKNETVDLEKIPIEEVFEQLKCSREGLTSDEGANRLQIFGPNKLEEKK    57
PHA4      ---MASNLSLEDIKNEQIDLENIPVEEVFQQLKCSKEGLSSAEGQKRVEIFGPNKLEEKK    57
PHA5      ---MAANLSLEELKNEKVDLESIPVEEVFQILKCSKEGLTKEEGQKRIEIFGPNKLEEKK    57
                        .: :***.**:::**: *:*..***.      :*: ::* ******.

PHA6      ESKFLNFLRYSWNPLSRTMVAAAIMAIVLANGGGRPPDWQLFLGIVTFLIVTNSSYTFVA    109
PHA7      ESKILKFLGFMWNPLSWVMEAAAIMALFLPHGKHKGVDYQDFVGIVALLIINS-TISFME    119
PHA1      ESKFLKFLGFMWNPLSWVMEAAAIMAIALANGGGKPPDWQDFVGIITLLVINS-TISFIE    117
PHA3      ESKFLKFLGFMWNPLSWVMEAAAIMAIALANGGGKPPDWQDFVGIITLLIINS-TISFIE    117
PHA2      ESKILKFLGFMWNPLSWVMEAAAIMAIALANGNGKPPDWQDFVGIVCLLVINS-TISFIE    116
PHA4      DNKLLKFLGFMWNPLSWVMECAAIMAIVLANGGGKPPDWPDFVGITVLLIINS-TISFIE    116
PHA5      ENKVLKFLGFMWNPLSWVMEAAAIMSIVLANGGGKPPDWPDFVGIMVLLVINS-TISFIE    116
          :.*.*:** : ***** .* .****:: * .*  :  *:  *:**  :*::.. : :*:

PHA6      VKLT-SDIAARVQFLHKFCRVLRHGRWCEVDSSNLVPGDIIHIKMGDIVPADVRILNFFT    168
PHA7      ENNAGNAAAALMARLAPKAKVLRDGKWNEEDAAVLVPGDIISIKLGDIIPADARLLN---    176
PHA1      ENNAGNAAAALMARLAPKAKVLRDGKWNEEDAAVLVPGDIISIKLGDIIPADARLLE---    174
PHA3      ENNAGNAAAALMARLAPKAKVLRDGKWDEEDAAVLVPGDIISIKLGDIIPADARLLE---    174
PHA2      ENNAGNAAAALMAGLAPKTKVLRDGRWSEQEAAILVPGDIISVKLGDIVPADARLLE---    173
PHA4      ENSAGNAASALMANLAPKTKILRDGKWSEEEASILVPGDIISIKLGDIVPADARLLE---    173
PHA5      ENNAGNAAAALMANLAPKTKVLRDGKWSEEDASLLVPGDLISVKLGDIIPADARLLE---    173
           : :  .  :* :  *    ::**.*:* * ::: *****:* :*:***:***.*:*:

PHA6      TDPVVKIDLSDLTGESLPVTKSRIGDCAYSGSTCQKGEIIAVVIAIGVRTRYRNAAAPHV    228
PHA7      -GDPLKIDQSALTGESLPVTKN-PGDGVYSGSTCKQGEIEAVVIATGVHTFFGKA--AHL    232
PHA1      -GDPLKIDQSALTGESLPVTKG-PGDGVYSGSTCKQGEIEAVVIATGVHTFFGKA--AHL    230
PHA3      -GDPLKIDQSALTGESLPVTKG-PGDGVYSGSTCKQGEIEAVVIATGVHTFFGKA--AHL    230
PHA2      -GDPLKIDQSALTGESLPVTKN-PGDEVFSGSTCKQGELEAVVIATGVHTFFGKA--AHL    229
PHA4      -GDPLKVDQAALTGESLPATKF-PGAEVFSGSTVKQGEIEAIVIATGVHTFFGKA--AHL    229
PHA5      -GDPLKIDQAALTGESLPVTKQ-PGDEVFSGSTVKQGELDAVVIATGVHTFFGKA--AHL    229
              :*:* : *******.**   *  .:**** ::**: *:*** **:* : :*   *:

PHA6      VKSSNQVECFRR----------------------------------VSKGIGNFCIHFVA    254
PHA7      VENTTHVGHFQK----------------------------------VLTSIGNFCICSIA    258
PHA1      VDSTNQVGHFQK----------------------------------VLTAIGNFCICSIA    256
PHA3      VDSTNQVGHFQKASRLFTLLFDAMIWINVSDFYEILHYKFMYGYRQVLTAIGNFCICSIA    290
PHA2      VDSTNNVGHFQK----------------------------------VLTAIGNFCICSIA    255
PHA4      VDSTNNVGHFQK----------------------------------VLTAIGNFCICSIA    255
PHA5      VDSTNQVGHFQK----------------------------------VLTAIGNFCICSIL    255
          *..:..*  *::                                  *  ..******  :

PHA6      VVMMVEIIVMYCIQHRAYRSGIDNLLVLLIGGIPIGMPLTLLLIMRHGTV--TSSHYVIK    312
PHA7      TGMIIELIVIFGGQHRHPREAVDSLLVLLIGGIPIAMPTVLSVTMAIGSHRLSQQGAITK    318
PHA1      VGMIIEIIVMYPIQHRKYRPGIDNLLVLLIGGIPIAMPTVLSVTMAIGSHRLAQQGAITK    316
PHA3      VGMIIEIIVMYPIQHRKYRPGIDNLLVLLIGGIPIAMPTVLSVTMAIGSHRLAQQGAITK    350
PHA2      VGMLIEIIVMYPIQHRKYRDGIDNLLVLLIGGIPIAMPTVLSVTMAIGSHRLSQQGAITK    315
PHA4      VGMVIEIVVMYPIQKRNYRDGIDNLLVLLIGGIPIAMPTVLSVTMAIGSHRLAQQGAITK    315
PHA5      VGIVIEIVVMWPIQKRKYRDGIDNLLVLLIGGIPIAMPTVLSVTMAIGSHRLSQQGAITK    315
          . :::*::*::  *:*  * .:*.***********.** .* : *  *:   :..  : *

PHA6      RMTTIIDMACMDVLCSDMTGTLTLGMLDVQRDLIEVFANDINKDTVALMAARACNSEECA    372
PHA7      RMTAIEEMAGMDVLCSDKTGTLTLNKLSVDKNLIEVFAKDVEKDMVVLMAARASRLENQD    378
PHA1      RMTAIEEMAGMDVLCSDKTGTLTLNKLTVDKNLVEVFAKGVDADTVVLMAARASRTENQD    376
PHA3      RMTAIEEMAGMDVLCSDKTGTLTLNKLTVDKALIEVFAKGIDADTVVLMAARASRIENQD    410
PHA2      RMTAIEEMAGMDVLCSDKTGTLTLNKLSVDKTLVEVFVKGVDKEYVLLLAARASRVENQD    375
PHA4      RMTAIEEMAGMDVLCSDKTGTLTLNKLTVDKNLIEVFPKDADKDTVMLLGARASRIENQD    375
PHA5      RMTAIEEMAGMDVLCSDKTGTLTLNKLTVDQTLIEVFPKNADADTVMLLAARASRVENQD    375
          ***:* :** ******* ******  * *:: *:*** :  : : * *:.***.. *:

PHA6      ---------------RRAGIS--LRLRCCPTEKQISHTYSDGKGKKHRVSIGKPEHILNH    415
PHA7      AIDTAIVSMLADPKEARAGITEVHFLPFNPTDKRTALTYLDSAGKMHRVSKGAPEQILNL    438
PHA1      AIDTAIVGMLSDPKEARAGIREIHFLPFNPTDKRTALTYLDGEGKMHRVSKGAPEQILNL    436
PHA3      AIDTAIVGMLADPQEARAGIREIHFLPFNPTDKRTALTYLDGEGKMHRVSKGAPEQILNL    470
PHA2      AIDACMVGMLADPKEARAGIREVHFLPFNPVDKRTALTYIDNNGNWHRASKGAPEQILDL    435
PHA4      AIDTCIVNMLGDPKEARAGIQEVHFLPFNPVEKRTAITYIDDKGNWHRASKGAPEQIIEL    435
PHA5      AIDACIVNMLGDAKLAREGIQEVHFFPFNPVDKRTAITYIDGNGDWHRASKGAPEQIIEL    435
                          * **     :   *.:*: : ** *  *. **.* * **:*::
```

```
PHA6            YAHNKSDIEHRVHSVINMFAEKGLKSMAVAYQEFPSDIEHRVHSAMDIFAEKVLHSIAEQ    475
PHA7            -AWNKSDIKNRVHSVIDKFAERGLRSLA--------------------------------    465
PHA1            -AHNKSDIERRVHAVIDKFAERGLRSLG--------------------------------    463
PHA3            -AHNKSDIERRVHTVIDKFAERGLRSLG--------------------------------    497
PHA2            -CNCKEDVRRKVHSMIDKYAERGLRSLA--------------------------------    462
PHA4            -CELKGDIKKKALEIIDDYANRGLRSLG--------------------------------    462
PHA5            -CGLSGSVLKKSHEIIDNFANRGLRSLG--------------------------------    462
                 .  . .: .:   :*: :*::**:*:.

PHA6            ANPSDIEQSVHSVIDMFAEKVLHSITLAFQEIPSDIKHTVRSMIDVFSEKVLHSIAEAYQ    535
PHA7            --------------------------------------------------------VARQ    469
PHA1            --------------------------------------------------------VAYQ    467
PHA3            --------------------------------------------------------VAYQ    501
PHA2            --------------------------------------------------------VARQ    466
PHA4            --------------------------------------------------------LARQ    466
PHA5            --------------------------------------------------------VARQ    466
                                                                         * *

PHA6            EVPHGSKEKSGSPWQLIGLLPFVDPPREDCASSIRELLNHGVNVKMITRDHMVIGKEIGR    595
PHA7            EVPEGTKDSPGGLWEFVGLLPLFDPPRHDSAETIRRALELGVSVKMITGDQLAIGKETGR    529
PHA1            EVPEGRKESSGGPWQFIGLLPLFDPPRHDSAETIRRALNLGVNVKMITGDQLAIGKETGR    527
PHA3            EVPEGRKESAGGPWQFIALLPLFDPPRHDSAETIRRALNLGVNVKMITGDQLAIGKETGR    561
PHA2            EVPEKSKESAGGPWQFVGLLPLFDPPRHDSAETIRRALNLGVNVKMITGDQLAIAKETGR    526
PHA4            TVPEKNKESEGSPWEFVGLLPLFDPPRHDSAETIRKALELGVAVKMITGDQLAIGKETAR    526
PHA5            TVPEKDKESAGSPWEFVGLLPLFDPPRHDSAETIRKALELGVNVKMITGDQLAIGKETGR    526
                 **.  *:. *. *:::.***:.****.*.*.:**. *: ** ***** *::.*.** .*

PHA6            RLGMGTNMYPSSALLGHNDKSIAADNDLSIDEIIENADGFVSCALPEQKYEIIKHLQSRK    655
PHA7            RLGMGTNMYPSSFLLGEQKDSSA--AVLPIEELIESADGFAG-VFPEHKYEIVRILQSRK    586
PHA1            RLGMGTNMYPSSALLGQTKDESI--ASLPIDELIEKADGFAG-VFPEHKYEIVKRLQARK    584
PHA3            RLGMGTNMYPSSALLGQTKDESI--AALPIDELIEKADGFAG-VFPEHKYEIVKRLQARK    618
PHA2            RLGMGTNMYPSASLLGQDKDSSI--ASLPVEELIEKADGFAG-VFPEHKYEIVKKLQERK    583
PHA4            RLGMGTNMYPSSALLGEHKDAAI--ASIPVDELIEKADGFAG-VFPEHKYEIVKKLQDMK    583
PHA5            RLGMGTNMYPSSALLGDHKDESI--AQIPVEELIEQADGFAG-VFPEHKYEIVKKLQERK    583
                ***********: ***. ..       : ::*:**.****.. .:**:****:: **  *

PHA6            HVCGMIGGGVDDIPALKKADIRIAPYGVTEAAQNAADIVLMESGLKYLCEAVLTSRSITV    715
PHA7            HICGMTGDGVNDAPALKKADIGIAVADATDAARGASDIVLTEPGLSVIIHAVLTSRAIFQ    646
PHA1            HICGMTGDGVNDAPALKKADIGIAVDDATDAARSASDIVLTEPGLSVIISAVLTSRAIFQ    644
PHA3            HICGMTGDGVNDAPALKKADIGIAVDDATDAARSASDIVLTEPGLSVIISAVLTSRAIFQ    678
PHA2            HIVGMTGDGVNDAPALKKADIGIAVADATDAARGASDIVLTEPGLSVIISAVLTSRAIFQ    643
PHA4            HICGMTGDGVNDAPALKKADIGIAVDDATDAARSASDIVLTEPGLSVIVSAVLTSRAIFQ    643
PHA5            HICGMTGDGVNDAPALKKADIGIAVADATDAARGASDIVLTEPGLGVIISAVLTSRAIFQ    643
                *: ** * **:* ******** **   .*:**: *:**** * **  :  ******:*

PHA6            RMKSCMVHAVSIAIHNVLGFMLLALVWKFDFPPFKMLIIALANCFINVIASQNRVKSSPL    775
PHA7            RMKNYTIYAVSITIRIVLGFMLLTAFWKFNFPPFMVLVIAILNDGTIMTISKDRVKPSPL    706
PHA1            RMKNYTIYAVSITIRIVLGFMLLALIWKFDFPPFMVLIIAILNDGTIMTISKDRVKPSPL    704
PHA3            RMKNYTIYAVSITIRIVLGFMLLALIWKFDFPPFMVLIIAILNDGTIMTISKDRVKPSPL    738
PHA2            RMKNYTIYAVSITIRIVFGFMLIALIWKYDFSAFMVLIIAILNDGTIMTISKDRVKPSPM    703
PHA4            RMKNYTIYAVSITIRVVMGFMLIALIWKFDFSPFMVLIIAILNDGTIMTISKDRVVPSPL    703
PHA5            RMKNYTIYAVSITIRVVMGFMLIALIWEFDFPPFMVLIIAILNDGTIMTISKDRVKPSPL    703
                ***.  ::****:*: *:****:: .*:::*  * :*:**: *    :  *::**  **:

PHA6            PVNWSLSEILTTGIVLGSYLAMSTVIFFWAVYETNFFSLDKT------------------    817
PHA7            PDSWKLSEIFAIGIVLGSYLALMTALFFYLTFETSFFANAFHVTDFNKHIPENKVITDSL    766
PHA1            PDSWKLAEIFTTGVVLGGYLAMMTVIFFWAAYETDFFPRVFGVSTLQRTATD-------D    757
PHA3            PDSWKLAEIFTTGVILGGYLAMMTVIFFWAAYKTNFFPRVFGVSTLEKTATD-------D    791
PHA2            PDSWKLNEIFATGVVLGGYQALMTVLFFWAMHDTKFFSDKFGVKDIRES-----------    752
PHA4            PDSWKLNEIFATGVVLGTYQAIMTVVFFYLAADTDFFTENFHVRSIRNS-----------    752
PHA5            PDSWKLNEIFATGIILGTYQALMTVLFFYLAASTDFFTEKFGVRSIREN-----------    752
                * .*.* **:: *::** * *: *.:**:   .*.**
```

```
PHA6      YMKFVLAVLLQVSSSNYALIFVTRTGKLL----PGCDSLLFIWVSYQLL-------FTML    866
PHA7      NAKLASAVYLQVSTISQALIFVTRSRGWSFMERPGLL-LVVAFIVAQLVATFMSAMVTSV    825
PHA1      FRKLASAIYLQVSTISQALIFVTRSRSWSFVERPGLL-LVVAFLIAQLVATLIA-VYASW    815
PHA3      FRKLASAIYLQVSTISQALIFVTRSRSWSFVERPGLL-LVFAFFVAQLVATLIA-VYANW    849
PHA2      DEEMMSALYLQVSIISQALIFVTRSRSWSFVERPGAL-LMIAFLIAQLVATLIA-VYADW    810
PHA4      PNELTAALYLQVSIISQALIFVTRSRSWSFVERPGLM-LVGAFFAAQLVATVLA-VYADW    810
PHA5      EHGLTAAVYLQVSIISQALIFVTRSRSWSFVERPGFL-LVVAFFLAQFVATLIT-VYANW    810
             :  *: ****  . *******:        **   *:  :.  *::        :

PHA6      AVSGDQSICWSCAGKIWLYNIVFYVPLYCIKFFNQRSRKGFLKSIL*-------------    912
PHA7      KFAGIEKIGWRWTGVIWLFNIITYFLLDPIKFAVRYALSGRAWGLLLNQKTAFTNRKDFG    885
PHA1      SFAAIEGIGWGWAGVIWLYNLVFYFPLDIIKFLIRYALSGRAWDLVLEQRIAFTRKKDFG    875
PHA3      SFAAIEGIGWGWAGVIWLYNIVTYIPLDLIKFLIRYALSGKAWDLVLEQRIAFTRKKDFG    909
PHA2      TFARVKGCGWGWAGVIWIFSIVTYFPLDIMKFAIRYILSGKAWNNLLDNKTAFTTKKDYG    870
PHA4      EFARIKGVGWGWAAVIWVYTIITYLPQDVLKFIIRFGLSGRAWDTMIQNKTAFTTKKDYG    870
PHA5      NFARIHGIGWGWAAIIWVYTIITYLPLDVLKFISRYALSGDAWDSMIQNKTAFTTKKDYG    870
           .:  .   *  :. **::.:: *.    :**  :   .*     :

PHA6      ------------------------------------------------------------    912
PHA7      KEAREAAWAAEQRTIHGLQSVET-KTFPENYTFREISVMAEEAKRRADIARLRELHTLKG    944
PHA1      KEQRELQWAHAQRTLHGLQVPDT-KLFSESTNFNELNQLAEEAKRRAEIARQRELHTLKG    934
PHA3      KELRELQWAHAQRTLHGLQVPDP-KIFSETTNFNELNQLAEEAKRRAEIARLRELHTLKG    968
PHA2      KEEREAQWALAQRTLHGLQPPEASNLFNEKNSYRELSEIAEQAKRRAEMARLRELHTLKG    930
PHA4      RGEREAQWALAQRTLHGLQTPEAAGLFNDK-NYRELSEIAEQAKRRAEVARLRELHTLKG    929
PHA5      KGEREAQWAVDQRTRHGLQTAESNGLFHDK-NYRELNEIAEQAKRRAEVAKYTHE*----    924


PHA6      -----------------------   912
PHA7      KVESFAKLRGLDVDHVNPHYTV*   966
PHA1      HVESVVKLKGLDIETIQQSYTV*   956
PHA3      HVESVVKLKGLDIETIQQSYTV*   990
PHA2      HVESVVKLKGLDIETIQQHYTV*   952
PHA4      HVESVVKLKGLDIETIQQHYTV*   951
PHA5      -----------------------   924
```

Fig. S1. Alignment of the protein sequences of the plasma membrane H^+-ATPases identified in *S. tuberosum* Phureja (PHA1: PGSC0003DMP400055772 PHA2: PGSC0003DMP400007331, PHA3: PGSC0003DMP400043938 PHA4: PGSC0003DMP400021001, PHA5: PGSC0003DMP400013900, PHA6: PGSC0003DMP400002571, PHA7: PGSC0003DMP400060260). Sequences were aligned with the Clustal Omega program (http://www.ebi.ac.uk/Tools/msa/clustalo/). Identities are indicated by asterisks below the sequence. Conserved substitutions are marked with two vertical dots and semi-conserved substitutions are marked with a single dot. Unaligned residues are shown as dashes within the sequence line. The putative transmembrane domains (identified using the Phobius program, EMBL, version 1.1, http://www.ebi.ac.uk/Tools/pfa/phobius/) are shaded in gray. The 14-3-3 binding site is marked in blue. The conserved sequences common to P-ATPases (TGES, DKTGTLT, GDGVNDA and KGAP) are indicate in red.

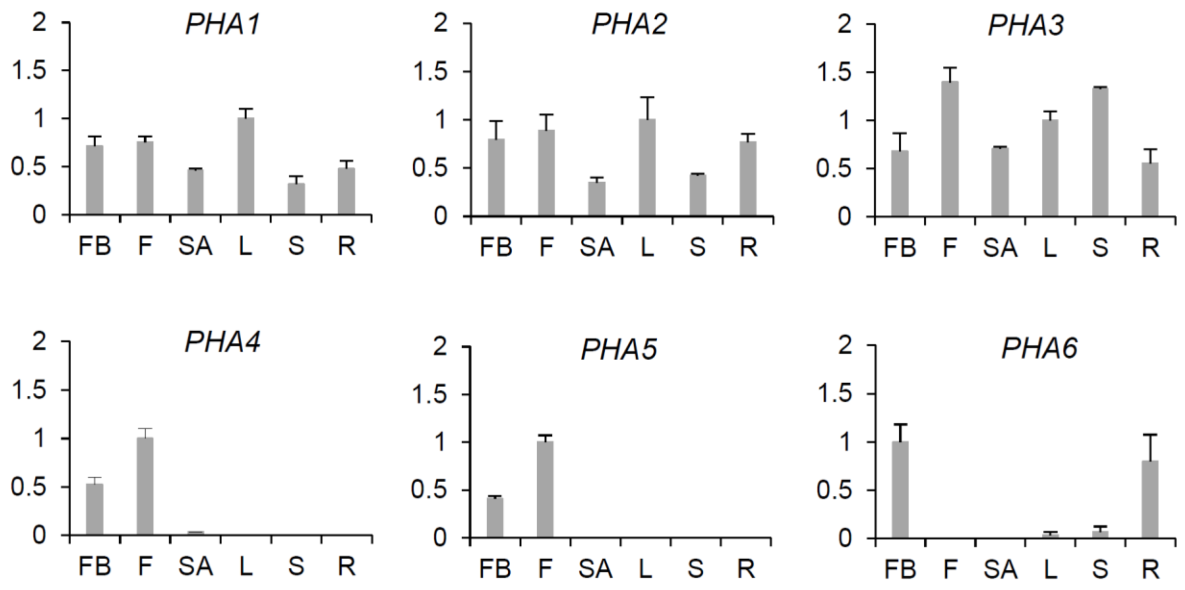

Fig. S2. Quantification of RT-PCR bands of Fig. 2A. Quantitative data of RT-PCR bands (mean ± SEM) of three independent experiments are displayed in the bar graph (arbitrary units). FB: flower bud, F: flower, SA: shoot apex, L: leaf, S: stem, R: root.

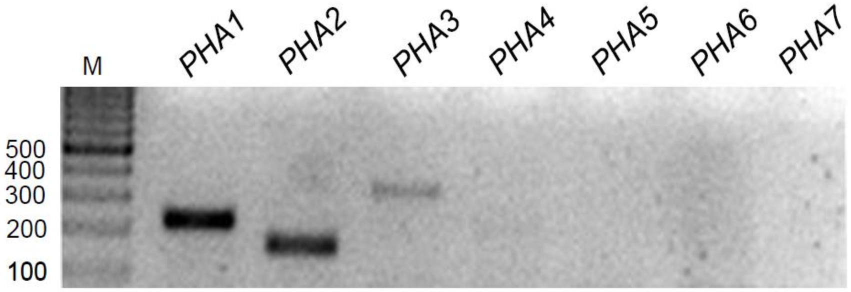

Fig. S3. RT-PCR analysis of PHA genes in stolons cultured *in vitro* under tuber-inducing conditions (MS medium plus 8% sucrose) for 15 days. The expected molecular weight of the amplicons are: 206 base pairs (bp) for PHA1, 141 bp for PHA2, 341 bp for PHA3, 372 bp for PHA4, 357 bp for PHA5, 203 bp for PHA6 and 339 bp for PHA7. M: molecular weight marker (numbers indicate the size of the DNA fragments in bp). A representative analysis of three independent experiments is shown.

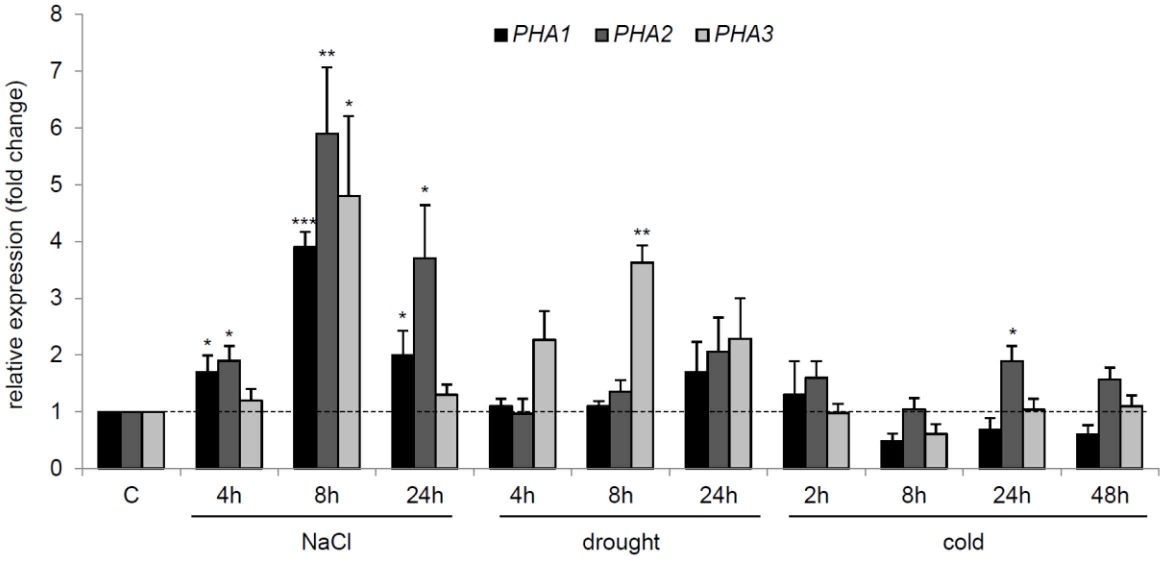

Fig. S4. Expression of PHA genes in response to abiotic stress. Detached leaves from soil-grown potato plants were treated with 250 mM NaCl or deprived of water (drought) for the indicated times. *In vitro*-grown potato plants were exposed to 4°C (cold) for the indicated times, while control plants remained at 22ºC. Total RNA was isolated from leaves and quantitative PCR was performed to determine the expression of PHA genes. Data are presented as expression level relative to the respective controls (C). Quantitative data (mean ± SEM) of three independent experiments, each consisting of four technical replicates, are displayed in the bar graph. The asterisks indicate statistical significance (*$p<0.05$, **$p<0.01$, ***$p<0.005$, with respect to controls).

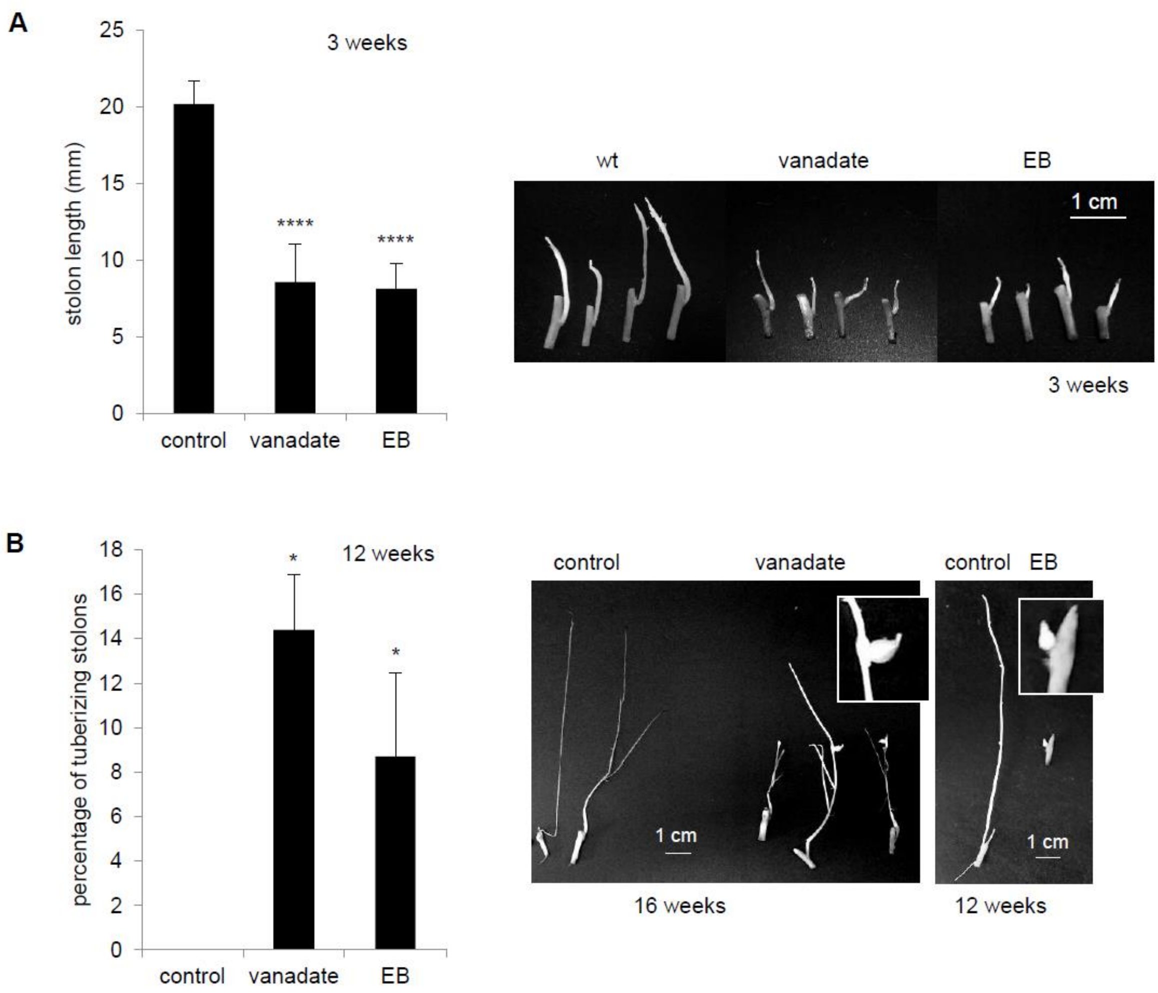

Fig. S5. Effect of vanadate and erythrosine B on stolons cultured under non-inducing conditions. Stolons were cultured under non-inducing conditions (MS medium plus 2% sucrose) in the absence (control) or presence of 1 mM vanadate or 50 μM erythrosine B (EB). (A) Stolon length, determined after 3 weeks of culture in the absence or presence of inhibitors; a representative image is shown. (B) The percentage of tuberizing stolons was determined after 12 weeks of culture in the absence or presence of inhibitors; a representative image is shown. Quantitative data of three independent experiments (mean ± SEM) are displayed in the bar graphs. The asterisks indicate statistical significance (*$p<0.05$, ****$p<0.001$, with respect to control). In these experiments performed under non-inducing conditions, the process was studied for 12-16 weeks, since tuber formation is significantly delayed with respect to tuber-inducing conditions.

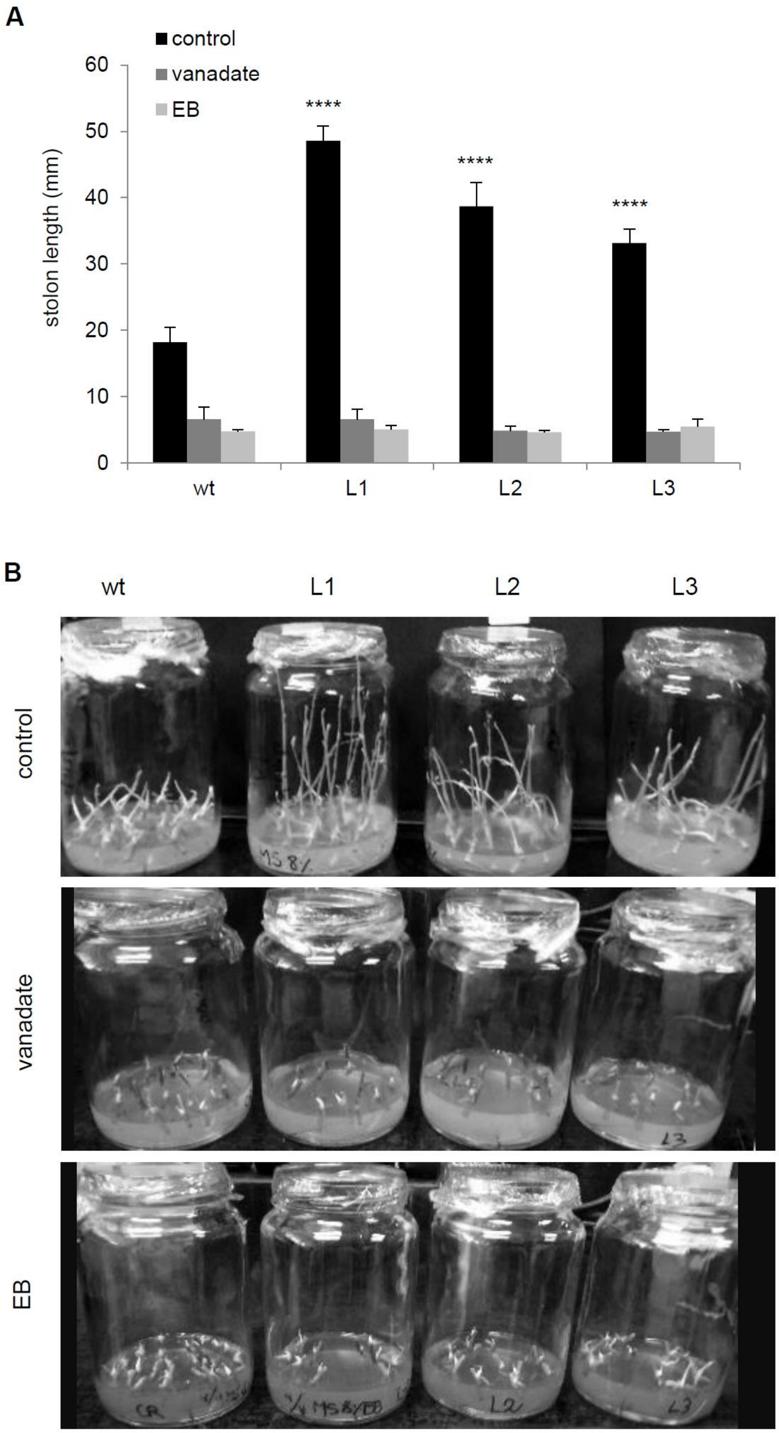

Fig. S6. Stolon length of PHA1-OE cultured in the presence of PM H^+-ATPase inhibitors. Stolons were cultured under tuber-inducing conditions (MS medium plus 8 % sucrose) for two weeks, in the absence or presence of 1 mM vanadate or 50 μM erythrosine B (EB). (A) Stolon length; data of three independent experiments (mean ± SEM; ****p<0.001, with respect to wt). (B) Representative image of stolons.

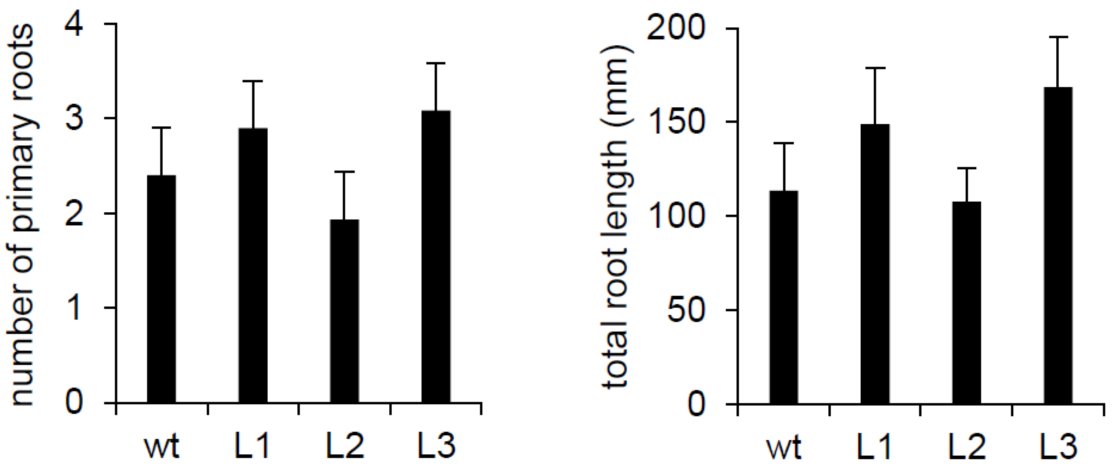

Fig. S7. Phenotypic analysis of PHA1-OE plants grown *in vitro*. Wild type (wt) and transgenic (L) plants generated from single-node cuttings were grown on MS medium plus 2% sucrose. After 2 weeks, the number of primary roots and total root length (calculated as the sum of the primary root lengths) were determined. Quantitative data of four independent experiments (mean ± SEM) are displayed in the bar graphs. No significant differences were detected between wt and transgenic lines (evaluated by Student's t test).

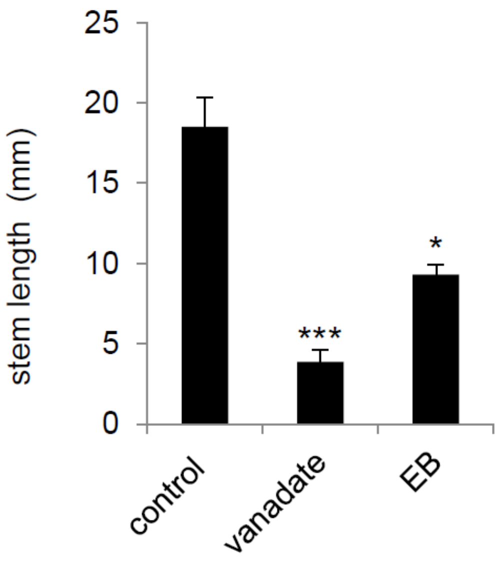

Fig. S8. Stem length of wild type plants generated from single-node cuttings, grown on MS medium plus 2 % sucrose in the absence (control) or presence of of 1 mM vanadate or 50 µM erythrosine B (EB). Quantitative data of three independent experiments (mean ± SEM) are displayed in the bar graph. The asterisks indicate statistical significance (*$p<0.05$, ***$p<0.005$, with respect to control).

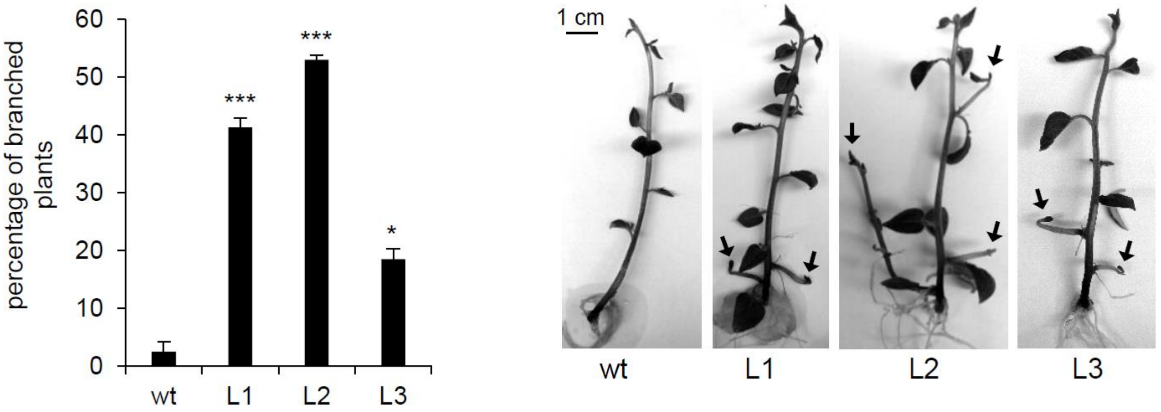

Fig. S9. Branching of PHA1-OE plants. Wild type (wt) and transgenic lines (L) generated from shoot apexes were grown on MS medium plus 2% sucrose for 3 weeks. The percentage of branched plants was determined as the number of plants presenting at least one branch equal or longer than 5 mm, with respect to the total number of plants (10-15 plants). A representative image of the plants is shown; arrows indicate branches. Quantitative data of three independent experiments (mean ± SEM) are displayed in the bar graph. The asterisks indicate statistical significance (* $p<0.05$, *** $p<0.005$, with respect to wt).
